# Supplementary material for: Differing impacts of global and regional responses on SARS-CoV-2 transmission cluster dynamics
Source: bioRxiv. 2020 Nov 6:2020.11.06.370999. Preprint. [Version 1] doi: 10.1101/2020.11.06.370999 (PMC7654859; doi:10.1101/2020.11.06.370999)
Supplement: 1 [file NIHPP2020.11.06.370999-supplement-1.pdf]

bioRxiv preprint doi: https://doi.org/10.1101/2020.11.06.370999; this version posted November 6, 2020. The copyright holder for this preprint (which was not certified by peer review) is the author/funder, who has granted bioRxiv a license to display the preprint in perpetuity. It is made available under aCC-BY 4.0 International license.

Supplementary Materials

Materials and Methods

Sequence data, metadata, and phylogenetic reconstruction A total of 11,262 sequences and associated metadata (time and country of sampling) were downloaded from GISAID (GISAID) on April 25, 2020. Sequence IDs can be found in Table S3 The number of confirmed cases for each corresponding country was retrieved from European Centre for Disease Prevention and Control. Cruise ships Diamond Princess and Grand Princess were treated as separate geographical regions for all analyses. Data regarding travel restrictions were obtained from WorldAware, COVID-19 Travel Restrictions Database, and Trip.com on June 4th, 2020; data on border closures from The New York Times on June 1st, 2020; airline restrictions from Bloomberg and Business Insider on June 4th, 2020. Data specifically for the Diamond Princess was retrieved from Business Insider on June 5th, 2020. Data on nationwide lockdowns, health screening measures, closure of non-essential business, the use of masks and restrictions of mass gatherings were obtained from different online newspapers, including CNN, South China Morning Post, The New York Times, The Miami Herald, and Reuters on June 2nd.

Sequences were quality filtered and aligned, as described in (*40*), keeping one representative per identical sequence group and excluding sequences without precise dates (month and day), resulting in $11,316$ sequences ($29,726$ nucleotides in length). We reconstructed a maximum likelihood tree with RAxML-NG (*41*) (GTR+I+G6 model, with all parameters optimized) starting from a distance tree (reconstructed using mimimum evolution in FASTME (*42*)), rooted it at the most recent common ancestor of the likely first-generation strains from (*1*), and put back the identical sequences (as zero-branch polytomies with the corresponding representative sequence tip). We then collapsed the branches without phylogenetic signal (i.e., of length $\leq 1/2$ mutation per genome). Support for branching events within the tree were calculated using the the

bioRxiv preprint doi: https://doi.org/10.1101/2020.11.06.370999; this version posted November 6, 2020. The copyright holder for this preprint (which was not certified by peer review) is the author/funder, who has granted bioRxiv a license to display the preprint in perpetuity. It is made available under aCC-BY 4.0 International license.

Shimodaira-Hasegawa approximate likelihood ratio test (*43*), performed in IQ-TREE v2 (*44*) on the final, fixed RAxML-NG tree topology.

Least squares dating in LSD2 (*45*) was used to date internal nodes of the tree (given sampling dates of taxa) and to identify, and remove, outlier sequences. Outlier sequences were defined as taxa whose mutation rate (estimated as the distance to the root divided by time since the first sequence) was larger than 3 standard deviations from the median. A strict molecular clock was assumed, resulting in an estimated mutation rate of $2.3[2.0-2.4] \cdot 10^4$ mutations per site per year.

Transmission cluster identification Clades comprised of ≥ 5 distinct sequences and a reliability of $\geq 90\%$ were recognized as potential transmission clusters when the median pairwise patristic distance (i.e., branch length separating sequences) within the clade was below a pre-specified percentile threshold of the whole-tree patristic distance distribution. A range of percentile thresholds spanning $0.0005\% - 25\%$ of the whole-tree distance distribution was used to choose an optimal threshold point and to verify robustness of cluster composition. The minimum percentile threshold that maximized the number of clusters was chosen as the optimal threshold by performing multiple clustering runs on randomly sampled patristic distance distributions (1 million for each run) in Phylopart v2 (*46*). Clusters and corresponding information (including sequence IDs) can be found in Table S4.

Potential clusters with more than two time points were used for the calculation of the basic reproductive number (R_0) from the viral effective population size (N_e) estimated using the skygrowth (*23*) package in R (*47*). Viral effective population size (N_e) estimates were allowed to vary weekly, and the default smoothing parameter (tau) of 0.1 was used. As described in Volz Didelot (2018) (*23*), the mean effective reproductive number (R_e) was calculated as a function of the change in Ne and 2-8 day (mean=5.475, SE=1.825) infectious period (psi). R_0

bioRxiv preprint doi: https://doi.org/10.1101/2020.11.06.370999; this version posted November 6, 2020. The copyright holder for this preprint (which was not certified by peer review) is the author/funder, who has granted bioRxiv a license to display the preprint in perpetuity. It is made available under aCC-BY 4.0 International license.

was defined as the first R_e value in time. Clusters with outlying R_0 values (> 3.4 standard deviations above or below the mean over all clusters) were discarded as unreliable.

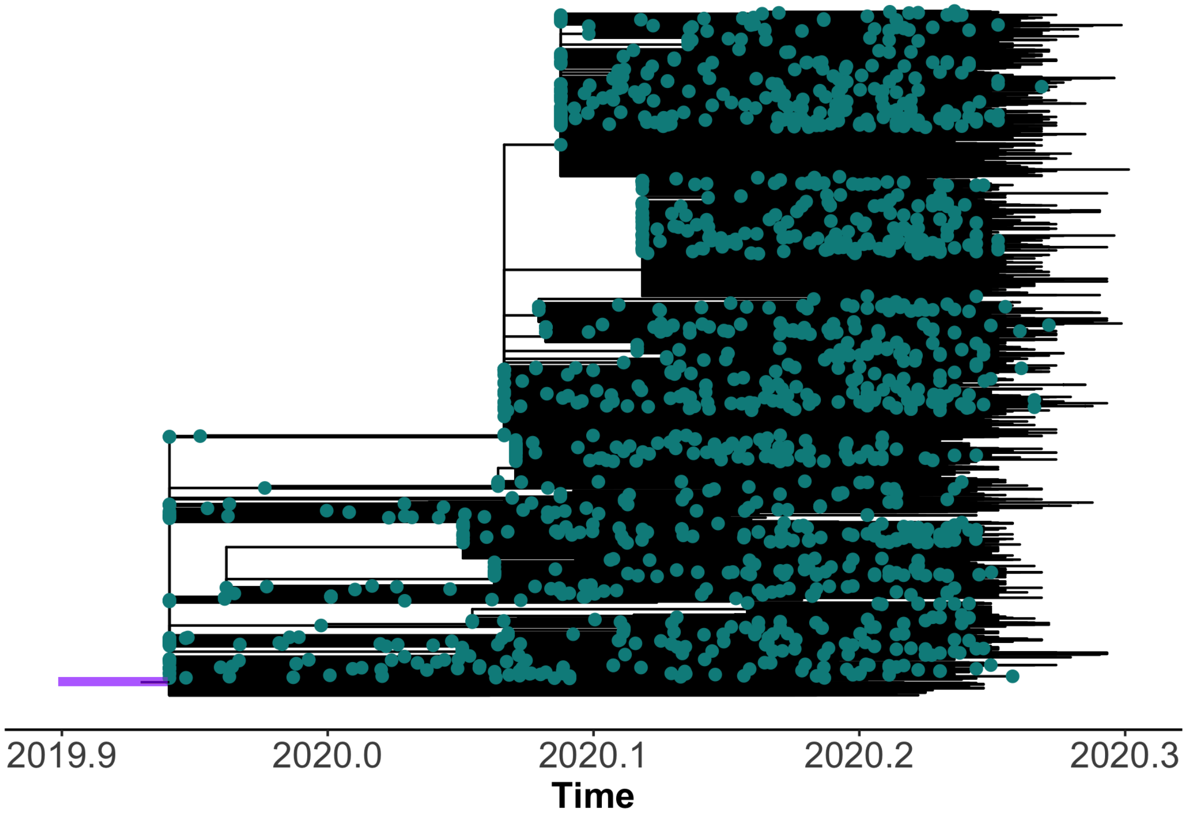

Figure S1: Maximum likelihood tree for 11,069 SARS-CoV-2 sequences scaled in time using least squares dating (*45*). Purple bar represents the 95% credible interval for estimate of the time to the most recent common ancestor for all sequences. Outlier sequences have been pruned and were excluded from downstream analyses (see Materials and Methods).

bioRxiv preprint doi: https://doi.org/10.1101/2020.11.06.370999; this version posted November 6, 2020. The copyright holder for this preprint (which was not certified by peer review) is the author/funder, who has granted bioRxiv a license to display the preprint in perpetuity. It is made available under aCC-BY 4.0 International license.

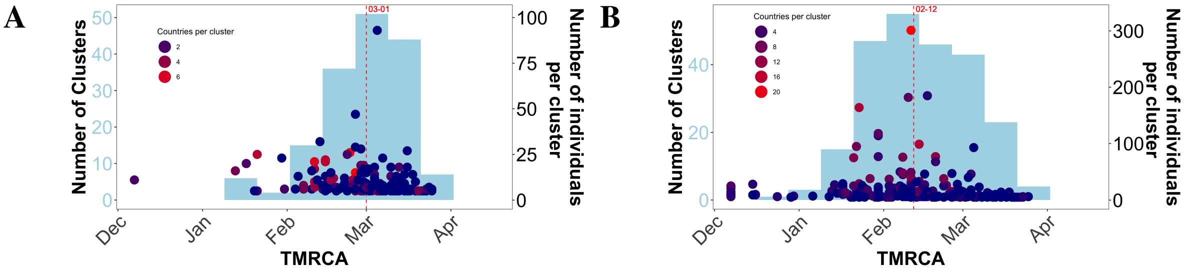

Figure S2: Timing of transmission cluster characteristics for additional distance thresholds defining transmission clusters. (A) Clusters with median patristic distances within 0.5% of the whole-tree patristic distance distribution. (B) Clusters with median patristic distances within 2.5% of the whole-tree patristic distance distribution. Bars represent the number of clusters (light blue) sharing a similar temporal origin, as inferred from the time to the most recent common ancestor (TMRCA) of corresponding cluster sequences. Dots correspond to the size (number of individuals) within the corresponding clusters at individual time points. Dots are colored according to the number of countries represented in each cluster. Red, dashed lines indicated median values.

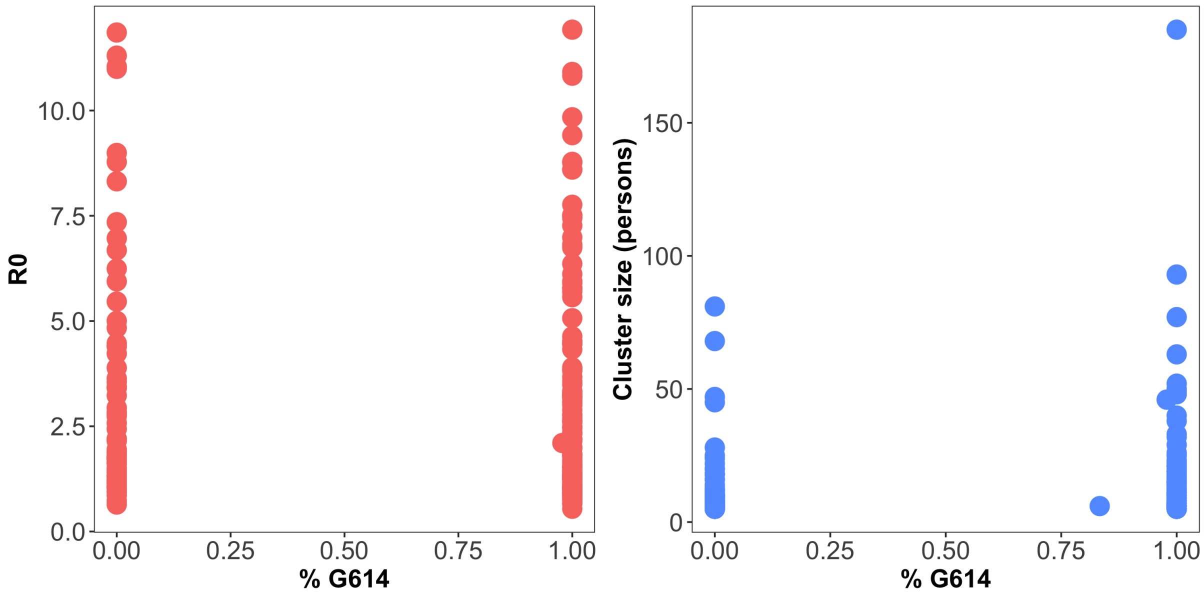

Figure S3: Relationship of R_0 (A) and (B) cluster size with fraction of individuals with D614G mutation for each cluster. Each dot represents an individual cluster. Clusters in (A) represent a subset of clusters in (B) (see Materials and Methods).

Table S1: Travel restriction data by country derived from various sources (see Materials and Methods)

Country	Lockdown	Travel ban entering from China	Travel ban entering from Europe	Travel ban entering from other countries	Non-citizens banned/ Travel across borders	Health screening measures at airports	Education closed or moved online	Non essential business closed	Masks	Mass gatherings
Australia	3/23/2020 nationwide	2/2/20	3/12/2020 from Italy	3/1/2020 Iran and South Korea	3/25/20	1/21/20	3/16/20	3/23/20		3/20/2020 over 100 people
Belgium	3/17/2020 nationwide				3/20/20	1/30/20	3/16/20	3/13/20	5/4/20	3/13/2020 all public gatherings
UK	3/23/2020 nationwide				3/17/20	1/30/20	3/18/20	3/21/20		3/17/20
US	03/19/2020 in California	2/2/20	3/13/2020 from Schengen area, Austria, Belgium 3/17/2020 from Ireland, UK	2/29/2020 from Iran 5/26/2020 from Brazil	3/21/2020 with Mexico and Canada	1/24/20	1/3/2020 in Florida	3/17/2020 in California	4/17/2020 in New York	3/11/2020 in Seattle over 250 people
Brazil	3/17/2020 in Santa Catarina	3/20/20	3/25/2020 from Norway, Schengen Area, Switzerland, UK,	3/25/2020 from Australia, Iceland, Japan, Korea (Rep.), Malaysia, Iran	3/30/20		3/25/20		4/22/20	
Canada					3/16/20	1/24/20	3/23/20	3/17/2020 in Ottawa	5/19/20	3/16/2020 over 50 people banned

bioRxiv preprint doi: https://doi.org/10.1101/2020.11.06.370999; this version posted November 6, 2020. The copyright holder for this preprint (which was not certified by peer review) is the author/funder, who has granted bioRxiv a license to display the preprint in perpetuity. It is made available under aCC-BY 4.0 International license.

Table S1: Travel restriction data by country derived from various sources (see Materials and Methods) .

Country	Lockdown	Travel ban entering from China	Travel ban entering from Europe	Travel ban entering from other countries	Non-citizens banned/ Travel across borders	Health screening measures at airports	Education closed or moved online	Non essential business closed	Masks	Mass gatherings
China	01/23/2020 in Wuhan	1/22/2020 ban outbound travel from Wuhan			3/26/20	1/21/20	2/4/20	1/25/20	2/8/2020 in Shanghai	4/1/2020 sports events
Netherlands		3/14/20	3/14/2020 from Italy	3/14/2020 from Iran, South Korea	3/17/20	1/30/20	3/17/20	3/15/20	6/1/20	3/12/2020 over 100 people
Dem Rep Congo	4/6/2020 in Gombe, Kinshasa and Goma				3/24/20	1/23/20	3/19/20	3/19/20	4/20/2020 in Kinshasa	3/19/2020 over 20 people
Finland					3/19/20	1/30/20	3/18/20	5/4/20		3/12/2020 over 500 people
France	3/17/2020 nationwide				3/17/20	1/30/20	3/16/20	3/14/20	5/10/20	3/13/2020 over 100 people
Germany	3/23/2020 nationwide				3/16/20	1/30/20	3/18/20	3/22/20	4/22/20	3/22/20
Iceland					3/20/20		3/12/20	3/23/20		3/16/2020 over 100 people

bioRxiv preprint doi: https://doi.org/10.1101/2020.11.06.370999; this version posted November 6, 2020. The copyright holder for this preprint (which was not certified by peer review) is the author/funder, who has granted bioRxiv a license to display the preprint in perpetuity. It is made available under aCC-BY 4.0 International license.

Table S1: Travel restriction data by country derived from various sources (see Materials and Methods)

Country	Lockdown	Travel ban entering from China	Travel ban entering from Europe	Travel ban entering from other countries	Non-citizens banned/ Travel across borders	Health screening measures at airports	Education closed or moved online	Non essential business closed	Masks	Mass gatherings
India	3/25/2020 nationwide	1/15/20	3/3/2020 travel from Italy	3/3/2020 from Iran, South Korea, Japan 2/15/2020 from Afghanistan, Austria, Belgium, Bulgaria, Croatia, Cyprus, Czechia, Denmark, Estonia, Finland, France, Germany, Greece, Hungary, Iceland, Ireland, Italy, Latvia, Liechten-stein, Lithuania, Luxem-bourg, Malaysia, Malta, Netherlands, Norway, Philippines, Poland, Portugal, Romania, Slovakia	3/22/20	1/29/20	3/25/20	3/23/20	4/9/2020 in Odisha	3/15/20

bioRxiv preprint doi: https://doi.org/10.1101/2020.11.06.370999; this version posted November 6, 2020. The copyright holder for this preprint (which was not certified by peer review) is the author/funder, who has granted bioRxiv a license to display the preprint in perpetuity. It is made available under aCC-BY 4.0 International license.

Table S1: Travel restriction data by country derived from various sources (see Materials and Methods).

Country	Lockdown	Travel ban entering from China	Travel ban entering from Europe	Travel ban entering from other countries	Non-citizens banned/ Travel across borders	Health screening measures at airports	Education closed or moved online	Non essential business closed	Masks	Mass gatherings
Italy	3/8/2020 nationwide	2/2/20			3/28/20	1/30/20	3/10/20	3/11/20	5/4/20	3/8/20

bioRxiv preprint doi: https://doi.org/10.1101/2020.11.06.370999; this version posted November 6, 2020. The copyright holder for this preprint (which was not certified by peer review) is the author/funder, who has granted bioRxiv a license to display the preprint in perpetuity. It is made available under aCC-BY 4.0 International license.

Table S1: Travel restriction data by country derived from various sources (see Materials and Methods)

Country	Lockdown	Travel ban entering from China	Travel ban entering from Europe	Travel ban entering from other countries	Non-citizens banned/ Travel across borders	Health screening measures at airports	Education closed or moved online	Non essential business closed	Masks	Mass gatherings
				3/26/2020 from Iran, Andorra, Austria, Belgium, Denmark, Estonia, France, Germany, Liechten-stein, Luxem-bourg, Malta, Monaco, Netherlands, Norway, Poland, San Marino, Slovenia, Sweden, Vatican City State 4/2/2020 from Albania, Armenia, Australia, Bolivia, Bosnia and Herzegov-ina, Brazil, Canada, Chile, Côte d'Ivoire (Ivory Coast), Dominica						

bioRxiv preprint doi: https://doi.org/10.1101/2020.11.06.370999; this version posted November 6, 2020. The copyright holder for this preprint (which was not certified by peer review) is the author/funder, who has granted bioRxiv a license to display the preprint in perpetuity. It is made available under aCC-BY 4.0 International license.

Table S1: Travel restriction data by country derived from various sources (see Materials and Methods)

Country	Lockdown	Travel ban entering from China	Travel ban entering from Europe	Travel ban entering from other countries	Non-citizens banned/ Travel across borders	Health screening measures at airports	Education closed or moved online	Non essential business closed	Masks	Mass gatherings
Luxembourg					3/16/2020 with Germany	1/24/20	3/16/20	3/16/20	4/20/20	3/13/2020 over 100 people
Mexico					3/21/2020 with USA	1/24/20	3/23/20	3/30/20		
Portugal	3/19/2020 nationwide		3/11/2020 from Italy3/18/2020 from Spain				3/16/20	3/13/20	5/4/20	3/15/2020 over 100 people
Russia	3/28/2020 nationwide	1/28/20	3/13/2020 Italy	3/4/2020 from Iran, South Korea	3/14/2020 with Poland and Norway	1/30/20	3/16/20	3/28/20	5/11/20	3/10/2020 over 5000 people in Moscow
Singapore	4/7/2020 nationwide	1/31/2020 2/3/2020 from Hong Kong, Macau	3/4/2020 from Italy, Iran, Korea (Rep) 3/15/2020 from France, Germany, and Spain					4/7/20	4/14/20	3/24/2020 over 10 people
Spain	3/14/2020 nationwide		3/13/2020 from Italy		3/17/20	1/30/20	3/16/20	3/14/20	5/4/20	2/12/2020 over 1000 people in Madrid
Sweden					3/19/20	1/30/20	3/18/2020 some locally			3/27/2020 over 50 people

bioRxiv preprint doi: https://doi.org/10.1101/2020.11.06.370999; this version posted November 6, 2020. The copyright holder for this preprint (which was not certified by peer review) is the author/funder, who has granted bioRxiv a license to display the preprint in perpetuity. It is made available under aCC-BY 4.0 International license.

Table S1: Travel restriction data by country derived from various sources (see Materials and Methods) .

Country	Lockdown	Travel ban entering from China	Travel ban entering from Europe	Travel ban entering from other countries	Non-citizens banned/ Travel across borders	Health screening measures at airports	Education closed or moved online	Non essential business closed	Masks	Mass gatherings
Switzerland		2/28/20	3/17/2020 with Italy, Austria, France, Germany		3/26/20		3/16/20	3/16/20		3/2/2020 over 1000 people
Taiwan		2/4/20			3/19/20	1/22/20	2/21/20		4/1/20	2/29/2020 in Taipei
Diamond Princess	2/1/2020 quarantined, docked in Okinawa									
Grand Princess	3/4/2020 CDC orders some passengers self-quarantine									

bioRxiv preprint doi: https://doi.org/10.1101/2020.11.06.370999; this version posted November 6, 2020. The copyright holder for this preprint (which was not certified by peer review) is the author/funder, who has granted bioRxiv a license to display the preprint in perpetuity. It is made available under aCC-BY 4.0 International license.

Table S2: Airline travel data obtained from various sources (see Materials and Methods).

Airline	Base	Date	Ban
Cabo Verde Airlines	Africa	3/18/20	Suspending all flights
SA express	Africa	3/18/20	Suspending all flights
Aerolineas Argentina	Argentina	3/18/20	Cut capacity to Rome, Madrid, Miami, New York and Orlando (various dates)
Qantas Airways	Australia	2/9/20	Flights to Beijing and Shanghai; cut capacity to Hong Kong, Sapporo, Auckland, Tokyo and Osaka
Tigerair Australia	Australia	3/31/20	Suspending all flights
Virgins Australia	Australia	2/11/20	Permanent cancellation of routes to Hong Kong from Melbourne (from Feb. 11) and Sydney
Austrian Airlines	Austria	1/29/20	Flights to Venice, Bologna, Milan, Beijing, Shanghai, Tel Aviv and Iran; cut capacity to European routes by 20%
Bahamas Air	Bahamas	3/23/20	Suspending all flights
Gulf Air	Bahrain	2/27/20	Flights to Dubai, Iraq and Lebanon; cut capacity to several other destinations
Air Antwerp	Belgium	3/22/20	Canceling flights until April 22
Brussels Airline	Belgium	3/12/20	All flights to and from Italy
LATAM Airlines Brasil	Brazil	3/2/20	Flights between Sao Paulo and Milan
Air Canada	Canada	1/30/20	All flights to and from mainland China and Italy; flights between Toronto and Hong Kong; maintaining flights between Vancouver and Hong Kong
Air Transat	Canada	3/13/20	Gradually suspending all flights
Harbour Air	Canada	3/27/20	Suspending all flights
Porter Airlines	Canada	3/21/20	Suspending all flights
Sunwing Airlines	Canada	3/17/20	Suspending all flights
Winair	Caribbean	3/19/20	Suspending all flights
Avianca	Colombia	3/25/20	Suspending all flights
CSA Czech Airlines	Czech	3/16/20	Suspending all flights
TAME	Ecuador	3/17/20	Suspending all flights
EgyptAir	Egypt	3/31/20	Suspending all flights
Ethiopian Airlines	Ethiopia	2/3/20	Reduced capacity and total number of flights to Beijing, Shanghai, Guangzhou and Hong Kong

bioRxiv preprint doi: https://doi.org/10.1101/2020.11.06.370999; this version posted November 6, 2020. The copyright holder for this preprint (which was not certified by peer review) is the author/funder, who has granted bioRxiv a license to display the preprint in perpetuity. It is made available under aCC-BY 4.0 International license.

Table S2: Airline travel data obtained from various sources (see Materials and Methods).

Airline	Base	Date	Ban
Finnair	Finland	3/6/20	All flights to mainland China; cut capacity to Guangzhou, Hong Kong, Osaka, Seoul, Milan; cut capacity across European network by 20%; new route to Busan postponed
Air France	France	1/30/20	All scheduled flights to mainland China, Hong Kong, Taipei, Italy, Tel Aviv; will gradually resume operations to Beijing, Shanghai, Hong Kong and Taipei after March 29; cut capacity to Seoul, Singapore, Japan, and across Europe
La Champagnie	France	3/18/20	Suspending all flights
Transavia	France	3/23/20	Suspending all flights
Lufthansa	Germany	1/30/20	Flights to Beijing, Shanghai, Nanjing, Shenyang, Qingdao, Tehran and Israel; cut capacity to Hong Kong, Seoul, Italy, various locations throughout Europe and domestically; cut flight capacity by up to 50%
Cathay Pacific	Hong Kong	1/30/20	Flights to Japan, Tel Aviv, Jeju, Busan, Kaohsiung, Taichung, Seoul, Washington, London Gatwick, Rome; cut capacity of flights to and from mainland China by 90%; cut global capacity by about 40%
Wizz Air	Hungary	3/11/20	All flights to Italy; flights between Luton and Tel Aviv; cut capacity to various destinations
Air India	India	1/31/20	Flights between Delhi and Shanghai, and between Delhi and Hong Kong
IndiGO	India	2/1/20	Flights between Bengaluru and Hong Kong, between Delhi and Chengdu, and between Kolkata and Guangzhou (various suspension dates)
Spice Jet	India	3/25/20	Suspending all flights
Vistara	India	3/25/20	Suspending all flights
Iran Air	Iran	3/8/20	Flights to Vienna, Stockholm and Gothenburg; temporarily suspended all flights to Europe
Aer Lingus	Ireland	3/11/20	All flights to and from Italy
Lauda	Ireland	3/16/20	Gradually suspending all flights
Ryanair	Ireland	3/17/20	All flights to and from Italy
El Al Israel	Israel	3/27/20	Suspending all flights
Neos	Italy	1/30/20	All flights to and from China

bioRxiv preprint doi: https://doi.org/10.1101/2020.11.06.370999; this version posted November 6, 2020. The copyright holder for this preprint (which was not certified by peer review) is the author/funder, who has granted bioRxiv a license to display the preprint in perpetuity. It is made available under aCC-BY 4.0 International license.

Table S2: Airline travel data obtained from various sources (see Materials and Methods).

Airline	Base	Date	Ban
All Nippon Airways	Japan	1/23/20	Flights to nine cities in China, including Beijing, Shanghai and Guangzhou, from Tokyo and Osaka; flights between Osaka and Hong Kong; cut capacity to four cities in China and various domestic routes
Japan Airlines	Japan	2/6/20	Flights from Haneda to Guangzhou, Narita to Hong Kong Kong and Honolulu, Tokyo to Beijing, Shanghai and Dalian; cut capacity to some South Korea, Taiwan and domestic routes
Royal Jordanian Airlines	Jordan	3/17/20	Suspending all flights
Air Astana	Kazakhstan	4/15/20	Restrict flights in the country
Qazaq Airlines	Kazakhstan	4/15/20	Suspending all flights
Kenya Airlines	Kenya	1/31/20	Flights to and from Guangzhou
Kuwait	Kuwait	3/6/20	Suspending all flights
Air Baltic	Latvia	3/17/20	Suspending all flights Latvia, Lithuania, and Estonia
Middle East Airlines	Lebanon	3/19/20	Suspending all flights
Luxair	Luxembourg	3/24/20	Suspending all flights
Air Madagascar	Madagascar	3/20/20	Suspending all flights
Air Asia	Malaysia	1/24/20	All flights to Wuhan and selected flights to mainland China; all flights between the Philippines and mainland China, Hong Kong and Macau
Air Malta	Malta	4/2/20	Suspending all flights
Air Moldova	Moldova	4/1/20	Suspending all flights
FlyOne	Moldova	3/17/20	Suspending all flights
Montenegro Airlines	Montenegro	3/16/20	Suspending all flights
Royal Air Maroc	Morocco	1/31/20	Direct flights between Casablanca and Beijing
KLM	Netherlands	1/30/20	All flights to Italy; flights to Chengdu, Hangzhou and Xiamen and to Beijing and Shanghai; cut capacity to Hong Kong
Air New Zealand	New Zealand	2/9/20	Flights between Auckland and Shanghai; cut capacity to Shanghai and Hong Kong

bioRxiv preprint doi: https://doi.org/10.1101/2020.11.06.370999; this version posted November 6, 2020. The copyright holder for this preprint (which was not certified by peer review) is the author/funder, who has granted bioRxiv a license to display the preprint in perpetuity. It is made available under aCC-BY 4.0 International license.

Table S2: Airline travel data obtained from various sources (see Materials and Methods).

Airline	Base	Date	Ban
Norwegian Air Shuttles	Norway	3/15/20	All flights to U.S. from Rome, Paris, Barcelona, Madrid, Amsterdam, Athens, Oslo; all flights to Italy; majority of flights to U.S. from Amsterdam and Stockholm; large share of flights within Scandinavia; maintaining London to U.S. route
Copa Airlines	Panama	3/23/20	Suspending all flights
Cebu Airline	Philippines	2/2/20	Flights to mainland China and Taiwan, cut capacity to Hong Kong and Macau
Philippine Airlines	Philippines	3/26/20	Suspending all flights
LOT Polish Airway	Poland	2/1/20	Flights to Beijing and Italy
TAP Air Portugal	Portugal	3/5/20	Cut 2,500 flights, mostly to European destinations like Italy, Spain and France
Qatar Airways	Qatar	2/3/20	All flights to mainland China
Blue Air	Romania	3/21/20	Suspending all flights
Rwandair	Rwanda	1/31/20	Flights between Kigali and Guangzhou
Saudia	Saudi Arabia	2/2/20	All flights to China
SAS	Scandinavia	1/31/20	Flights to Beijing and Shanghai (from Jan. 31 to Apr. 30); flights to Bologna, Milan, Turin and Venice
Air Serbia	Serbia and Bosnia	3/19/20	Suspending all flights
Air Seychelles	Seychelles	3/9/20	Flights to Tel Aviv, Mumbai, Johannesburg and Mauritius
Jetstar Airways	Singapore	3/23/20	Suspending all flights
Scoot	Singapore	2/23/20	Flights to 19 cities to China including Wuhan, Guangzhou and Xi'an; ad-hoc cancellations of flights to Hong Kong and Macau
SilkAir	Singapore	2/4/20	Flights to Shenzhen, Xiamen, Chongqing and Hiroshima
Singapore Airlines	Singapore	2/3/20	Flights to Beijing, Shanghai and Guangzhou; cut capacity to Hong Kong, Los Angeles, London, Paris and dozens of other cities
Airlink	South Africa	3/28/20	Suspending all flights

bioRxiv preprint doi: https://doi.org/10.1101/2020.11.06.370999; this version posted November 6, 2020. The copyright holder for this preprint (which was not certified by peer review) is the author/funder, who has granted bioRxiv a license to display the preprint in perpetuity. It is made available under aCC-BY 4.0 International license.

Table S2: Airline travel data obtained from various sources (see Materials and Methods).

Airline	Base	Date	Ban
Kulula	South Africa	3/26/20	Suspending all flights
Air Seoul	South Korea	1/28/20	Flights between Incheon and Zhangjiajie and Linyi in China; between Incheon and Hanoi
Asiana Airlines	South Korea	2/4/20	Flights to dozens of Asian cities including Beijing, Guangzhou and Hong Kong; between Daegu and Jeju; flights to Venice, Rome and Barcelona; cut capacity to 15 cities, including Shanghai and Shenzhen (various suspension dates)
Korean Air	South Korea	1/24/20	Flights between Incheon and Wuhan, Huangshan, Zhangjiajie, Changsha, Kunming, Tel Aviv, Daegu; to Hanoi, Danang, Phu Quoc, Bangkok, Siem Reap, Taipei, Chubu, Aomori and Ulaanbaatar; between Busan and Beijing/Nanjing; between Jeju and Daegu. Also reduced frequency to 15 more routes
T'way Air	South Korea	2/5/20	Flights between South Korea and Hanoi
Iberia	Spain	1/31/20	Flights between Madrid and Shanghai
Swiss International Airlines	Switzerland	1/29/20	Flights to Beijing and Shanghai
China Airlines	Taiwan	1/23/20	Flights to and from Wuhan
Starlux	Taiwan	3/21/20	Suspending all flights
Thai Lion Air	Thailand	3/25/20	Suspending all flights
Pegasus Airlines	Turkey	2/25/20	Flights between Istanbul and Tehran
Sunexpress	Turkey	3/27/20	Suspending all flights
Turkish Airlines	Turkey	1/31/20	Flights to mainland China and Iran, except Tehran, Nakhcevan, Azerbaijan; cut capacity to Israel, Seoul and Nigeria
Turkmenistan Airlines	Turkmenistan	2/1/20	Flights to Beijing
Emirates	UAE	3/25/20	Suspending all flights

bioRxiv preprint doi: https://doi.org/10.1101/2020.11.06.370999; this version posted November 6, 2020. The copyright holder for this preprint (which was not certified by peer review) is the author/funder, who has granted bioRxiv a license to display the preprint in perpetuity. It is made available under aCC-BY 4.0 International license.

Table S2: Airline travel data obtained from various sources (see Materials and Methods).

Airline	Base	Date	Ban
Etihad Airways	UAE	2/5/20	Flights to Rome, Milan, Shanghai and Chengdu; maintaining service to Beijing
Fly Dubai	UAE	3/25/20	Suspending all flights
British Airways	UK	1/29/20	All flights to Italy, Beijing and Shanghai; cut capacity to New York, Singapore, France, Austria, Belgium, Germany, Ireland, Switzerland and Albania
Cayman Airline	UK	3/22/20	Suspending all flights
Comair British Airlines	UK	3/26/20	Suspending all flights
easyJet	UK	3/24/20	Suspending all flights
Jet2Go.com	UK	3/17/20	Suspending all flights
Virgina Atlantic	UK	2/1/20	Flights to Shanghai; maintaining Hong Kong service; new Sao Paulo route postponed
Ukraines International Airlines	Ukraine	3/17/20	Suspending all flights
Air Arabia	United Arab Emirates	3/25/20	Banned passengers from entering
American Airlines	USA	1/31/20	All flights to China and Argentina; Hong Kong service to Dallas and Los Angeles; Seoul service to Dallas; Milan service to New York and Miami; Rome service to Philadelphia, Chicago and Charlotte; Santiago service to Dallas; cut capacity to Paris, Madrid, Montevideo, Barcelona, Venice; cut summer international capacity by half
Delta	USA	2/2/20	All flights to China and Italy; cut capacity to South Korea, Japan, EU countries and Latin America
United Airlines	USA	2/5/20	Flights to Beijing, Shanghai, Chengdu and Hong Kong; also flights from Los Angeles, Houston and Chicago to Tokyo Narita; reduced capacity and schedule for flights from Newark, Honolulu and San Francisco to Tokyo Narita, Osaka Kansai, Singapore, Seoul and Taipei
Uzbekistan Airways	Uzbekistan	3/17/20	Suspending all flights

bioRxiv preprint doi: https://doi.org/10.1101/2020.11.06.370999; this version posted November 6, 2020. The copyright holder for this preprint (which was not certified by peer review) is the author/funder, who has granted bioRxiv a license to display the preprint in perpetuity. It is made available under aCC-BY 4.0 International license.

Table S2: Airline travel data obtained from various sources (see Materials and Methods).

Airline	Base	Date	Ban
VietJet Air	Vietnam	2/1/20	All flights to mainland China and South Korea; maintaining services to Hong Kong and Taiwan
Vietnam Airlines	Vietnam	2/4/20	Flights between Vietnam and Beijing, Shanghai, Guangzhou, Shenzhen, Chengdu, Macau and South Korean destinations; between Hanoi and Hong Kong; cut capacity to London, Paris, Frankfurt, Hong Kong and Seoul
Yemenia	Yemen	3/18/20	Suspending all flights
FastJet Zimbabwe	Zimbabwe	3/27/20	Suspending all flights

bioRxiv preprint doi: https://doi.org/10.1101/2020.11.06.370999; this version posted November 6, 2020. The copyright holder for this preprint (which was not certified by peer review) is the author/funder, who has granted bioRxiv a license to display the preprint in perpetuity. It is made available under aCC-BY 4.0 International license.

bioRxiv preprint doi: https://doi.org/10.1101/2020.11.06.370999; this version posted November 6, 2020. The copyright holder for this preprint (which was not certified by peer review) is the author/funder, who has granted bioRxiv a license to display the preprint in perpetuity. It is made available under aCC-BY 4.0 International license.

Table S3: GISAID sequence IDs collected on April 25, 2020.

ID
EPI_ISL_417541
EPI_ISL_419448
EPI_ISL_424900
EPI_ISL_419301
EPI_ISL_428832
EPI_ISL_413850
EPI_ISL_419738
EPI_ISL_424528
EPI_ISL_415529
EPI_ISL_421922
EPI_ISL_421900
EPI_ISL_427334
EPI_ISL_414562
EPI_ISL_404227
EPI_ISL_422032
EPI_ISL_417234
EPI_ISL_426763
EPI_ISL_420731
EPI_ISL_417386
EPI_ISL_417267
EPI_ISL_422888
EPI_ISL_429882
EPI_ISL_419824
EPI_ISL_424227
EPI_ISL_426307
EPI_ISL_425762
EPI_ISL_417813
EPI_ISL_422602
EPI_ISL_427208
EPI_ISL_424092
EPI_ISL_421934
EPI_ISL_423205
EPI_ISL_422019
EPI_ISL_421746
EPI_ISL_420003
EPI_ISL_424215
EPI_ISL_421411
EPI_ISL_416718

bioRxiv preprint doi: https://doi.org/10.1101/2020.11.06.370999; this version posted November 6, 2020. The copyright holder for this preprint (which was not certified by peer review) is the author/funder, who has granted bioRxiv a license to display the preprint in perpetuity. It is made available under aCC-BY 4.0 International license.

Table S3: GISAID sequence IDs collected on April 25, 2020.

ID
EPI_ISL_417669
EPI_ISL_402129
EPI_ISL_425857
EPI_ISL_426978
EPI_ISL_423952
EPI_ISL_420773
EPI_ISL_423332
EPI_ISL_419436
EPI_ISL_420691
EPI_ISL_420516
EPI_ISL_426689
EPI_ISL_429483
EPI_ISL_420174
EPI_ISL_419674
EPI_ISL_429022
EPI_ISL_429089
EPI_ISL_420569
EPI_ISL_423642
EPI_ISL_426852
EPI_ISL_422042
EPI_ISL_423384
EPI_ISL_425872
EPI_ISL_426979
EPI_ISL_417466
EPI_ISL_422071
EPI_ISL_428319
EPI_ISL_423221
EPI_ISL_422421
EPI_ISL_419907
EPI_ISL_427381
EPI_ISL_424498
EPI_ISL_427764
EPI_ISL_418433
EPI_ISL_426642
EPI_ISL_417348
EPI_ISL_424379
EPI_ISL_430097
EPI_ISL_419582

bioRxiv preprint doi: https://doi.org/10.1101/2020.11.06.370999; this version posted November 6, 2020. The copyright holder for this preprint (which was not certified by peer review) is the author/funder, who has granted bioRxiv a license to display the preprint in perpetuity. It is made available under aCC-BY 4.0 International license.

Table S3: GISAID sequence IDs collected on April 25, 2020.

ID
EPI_ISL_424482
EPI_ISL_428262
EPI_ISL_417092
EPI_ISL_429131
EPI_ISL_423161
EPI_ISL_419585
EPI_ISL_418885
EPI_ISL_421314
EPI_ISL_417332
EPI_ISL_418823
EPI_ISL_426941
EPI_ISL_416589
EPI_ISL_426085
EPI_ISL_423310
EPI_ISL_423375
EPI_ISL_418290
EPI_ISL_419935
EPI_ISL_417878
EPI_ISL_420478
EPI_ISL_421879
EPI_ISL_414526
EPI_ISL_419902
EPI_ISL_428272
EPI_ISL_422977
EPI_ISL_417181
EPI_ISL_425048
EPI_ISL_411952
EPI_ISL_427497
EPI_ISL_421427
EPI_ISL_427199
EPI_ISL_425596
EPI_ISL_415532
EPI_ISL_425238
EPI_ISL_420114
EPI_ISL_419532
EPI_ISL_425056
EPI_ISL_419712
EPI_ISL_420612

bioRxiv preprint doi: https://doi.org/10.1101/2020.11.06.370999; this version posted November 6, 2020. The copyright holder for this preprint (which was not certified by peer review) is the author/funder, who has granted bioRxiv a license to display the preprint in perpetuity. It is made available under aCC-BY 4.0 International license.

Table S3: GISAID sequence IDs collected on April 25, 2020.

ID
EPI_ISL_423000
EPI_ISL_416711
EPI_ISL_417514
EPI_ISL_416482
EPI_ISL_418641
EPI_ISL_421479
EPI_ISL_418033
EPI_ISL_422081
EPI_ISL_424032
EPI_ISL_421004
EPI_ISL_419265
EPI_ISL_425694
EPI_ISL_424476
EPI_ISL_429155
EPI_ISL_413853
EPI_ISL_417524
EPI_ISL_424050
EPI_ISL_429067
EPI_ISL_426729
EPI_ISL_425242
EPI_ISL_416365
EPI_ISL_417301
EPI_ISL_420847
EPI_ISL_422330
EPI_ISL_421260
EPI_ISL_421630
EPI_ISL_425206
EPI_ISL_421738
EPI_ISL_423474
EPI_ISL_426954
EPI_ISL_419559
EPI_ISL_426774
EPI_ISL_417663
EPI_ISL_420749
EPI_ISL_422889
EPI_ISL_427488
EPI_ISL_420477
EPI_ISL_426811

bioRxiv preprint doi: https://doi.org/10.1101/2020.11.06.370999; this version posted November 6, 2020. The copyright holder for this preprint (which was not certified by peer review) is the author/funder, who has granted bioRxiv a license to display the preprint in perpetuity. It is made available under aCC-BY 4.0 International license.

Table S3: GISAID sequence IDs collected on April 25, 2020.

ID
EPI_ISL_424221
EPI_ISL_427263
EPI_ISL_406531
EPI_ISL_416699
EPI_ISL_421617
EPI_ISL_418896
EPI_ISL_425383
EPI_ISL_418967
EPI_ISL_423477
EPI_ISL_417690
EPI_ISL_429162
EPI_ISL_422927
EPI_ISL_416676
EPI_ISL_427160
EPI_ISL_419499
EPI_ISL_417937
EPI_ISL_418894
EPI_ISL_427088
EPI_ISL_417224
EPI_ISL_424317
EPI_ISL_417850
EPI_ISL_425535
EPI_ISL_427247
EPI_ISL_425688
EPI_ISL_420360
EPI_ISL_420549
EPI_ISL_417222
EPI_ISL_417007
EPI_ISL_426092
EPI_ISL_415538
EPI_ISL_418931
EPI_ISL_421388
EPI_ISL_423135
EPI_ISL_426965
EPI_ISL_428233
EPI_ISL_422397
EPI_ISL_424546
EPI_ISL_424642

bioRxiv preprint doi: https://doi.org/10.1101/2020.11.06.370999; this version posted November 6, 2020. The copyright holder for this preprint (which was not certified by peer review) is the author/funder, who has granted bioRxiv a license to display the preprint in perpetuity. It is made available under aCC-BY 4.0 International license.

Table S3: GISAID sequence IDs collected on April 25, 2020.

ID
EPI_ISL_429491
EPI_ISL_421691
EPI_ISL_429782
EPI_ISL_419561
EPI_ISL_425988
EPI_ISL_419936
EPI_ISL_422939
EPI_ISL_423638
EPI_ISL_429531
EPI_ISL_424301
EPI_ISL_427290
EPI_ISL_422913
EPI_ISL_425904
EPI_ISL_415595
EPI_ISL_421002
EPI_ISL_416444
EPI_ISL_422128
EPI_ISL_421560
EPI_ISL_422527
EPI_ISL_419749
EPI_ISL_417122
EPI_ISL_422567
EPI_ISL_419834
EPI_ISL_423435
EPI_ISL_417144
EPI_ISL_415743
EPI_ISL_429504
EPI_ISL_427549
EPI_ISL_429744
EPI_ISL_418965
EPI_ISL_422667
EPI_ISL_417163
EPI_ISL_417350
EPI_ISL_422839
EPI_ISL_427773
EPI_ISL_420236
EPI_ISL_429717
EPI_ISL_427744

bioRxiv preprint doi: https://doi.org/10.1101/2020.11.06.370999; this version posted November 6, 2020. The copyright holder for this preprint (which was not certified by peer review) is the author/funder, who has granted bioRxiv a license to display the preprint in perpetuity. It is made available under aCC-BY 4.0 International license.

Table S3: GISAID sequence IDs collected on April 25, 2020.

ID
EPI_ISL_423968
EPI_ISL_425362
EPI_ISL_422747
EPI_ISL_415650
EPI_ISL_422917
EPI_ISL_420346
EPI_ISL_417022
EPI_ISL_417364
EPI_ISL_416450
EPI_ISL_427474
EPI_ISL_427353
EPI_ISL_423096
EPI_ISL_423063
EPI_ISL_423125
EPI_ISL_422949
EPI_ISL_427110
EPI_ISL_420527
EPI_ISL_418388
EPI_ISL_416698
EPI_ISL_424609
EPI_ISL_418770
EPI_ISL_414486
EPI_ISL_428254
EPI_ISL_418192
EPI_ISL_422880
EPI_ISL_425055
EPI_ISL_418114
EPI_ISL_423854
EPI_ISL_427787
EPI_ISL_426502
EPI_ISL_429281
EPI_ISL_425820
EPI_ISL_419173
EPI_ISL_430021
EPI_ISL_425682
EPI_ISL_420585
EPI_ISL_413653
EPI_ISL_430070

bioRxiv preprint doi: https://doi.org/10.1101/2020.11.06.370999; this version posted November 6, 2020. The copyright holder for this preprint (which was not certified by peer review) is the author/funder, who has granted bioRxiv a license to display the preprint in perpetuity. It is made available under aCC-BY 4.0 International license.

Table S3: GISAID sequence IDs collected on April 25, 2020.

ID
EPI_ISL_424342
EPI_ISL_426306
EPI_ISL_425430
EPI_ISL_424574
EPI_ISL_421289
EPI_ISL_420611
EPI_ISL_414366
EPI_ISL_426617
EPI_ISL_421207
EPI_ISL_420532
EPI_ISL_418294
EPI_ISL_419832
EPI_ISL_421281
EPI_ISL_417361
EPI_ISL_423176
EPI_ISL_417641
EPI_ISL_424499
EPI_ISL_414621
EPI_ISL_423379
EPI_ISL_418971
EPI_ISL_426993
EPI_ISL_428757
EPI_ISL_419460
EPI_ISL_424272
EPI_ISL_414043
EPI_ISL_428468
EPI_ISL_428232
EPI_ISL_418260
EPI_ISL_419568
EPI_ISL_429564
EPI_ISL_421473
EPI_ISL_427365
EPI_ISL_423823
EPI_ISL_425951
EPI_ISL_424354
EPI_ISL_424103
EPI_ISL_424960
EPI_ISL_417584

bioRxiv preprint doi: https://doi.org/10.1101/2020.11.06.370999; this version posted November 6, 2020. The copyright holder for this preprint (which was not certified by peer review) is the author/funder, who has granted bioRxiv a license to display the preprint in perpetuity. It is made available under aCC-BY 4.0 International license.

Table S3: GISAID sequence IDs collected on April 25, 2020.

ID
EPI_ISL_422323
EPI_ISL_420058
EPI_ISL_420208
EPI_ISL_423124
EPI_ISL_427084
EPI_ISL_425673
EPI_ISL_417481
EPI_ISL_423074
EPI_ISL_417536
EPI_ISL_420248
EPI_ISL_420209
EPI_ISL_413607
EPI_ISL_420483
EPI_ISL_429359
EPI_ISL_423936
EPI_ISL_417193
EPI_ISL_417477
EPI_ISL_418834
EPI_ISL_423560
EPI_ISL_416488
EPI_ISL_424449
EPI_ISL_417246
EPI_ISL_425521
EPI_ISL_427555
EPI_ISL_424162
EPI_ISL_425135
EPI_ISL_412898
EPI_ISL_421855
EPI_ISL_422303
EPI_ISL_429464
EPI_ISL_426555
EPI_ISL_426964
EPI_ISL_418796
EPI_ISL_418240
EPI_ISL_420461
EPI_ISL_429969
EPI_ISL_416368
EPI_ISL_427205

bioRxiv preprint doi: https://doi.org/10.1101/2020.11.06.370999; this version posted November 6, 2020. The copyright holder for this preprint (which was not certified by peer review) is the author/funder, who has granted bioRxiv a license to display the preprint in perpetuity. It is made available under aCC-BY 4.0 International license.

Table S3: GISAID sequence IDs collected on April 25, 2020.

ID
EPI_ISL_426685
EPI_ISL_426014
EPI_ISL_418218
EPI_ISL_413858
EPI_ISL_427786
EPI_ISL_420798
EPI_ISL_424956
EPI_ISL_419984
EPI_ISL_419899
EPI_ISL_425984
EPI_ISL_429485
EPI_ISL_429282
EPI_ISL_421291
EPI_ISL_415710
EPI_ISL_429467
EPI_ISL_418346
EPI_ISL_425913
EPI_ISL_414633
EPI_ISL_420460
EPI_ISL_429097
EPI_ISL_415479
EPI_ISL_429142
EPI_ISL_417816
EPI_ISL_417836
EPI_ISL_414539
EPI_ISL_426288
EPI_ISL_416427
EPI_ISL_418652
EPI_ISL_425891
EPI_ISL_422651
EPI_ISL_429082
EPI_ISL_420631
EPI_ISL_419564
EPI_ISL_421355
EPI_ISL_421450
EPI_ISL_426471
EPI_ISL_407976
EPI_ISL_418284

bioRxiv preprint doi: https://doi.org/10.1101/2020.11.06.370999; this version posted November 6, 2020. The copyright holder for this preprint (which was not certified by peer review) is the author/funder, who has granted bioRxiv a license to display the preprint in perpetuity. It is made available under aCC-BY 4.0 International license.

Table S3: GISAID sequence IDs collected on April 25, 2020.

ID
EPI_ISL_428373
EPI_ISL_427789
EPI_ISL_427650
EPI_ISL_420120
EPI_ISL_429511
EPI_ISL_419584
EPI_ISL_424880
EPI_ISL_419720
EPI_ISL_429305
EPI_ISL_414586
EPI_ISL_421417
EPI_ISL_422427
EPI_ISL_418646
EPI_ISL_418159
EPI_ISL_428359
EPI_ISL_423347
EPI_ISL_422871
EPI_ISL_419767
EPI_ISL_426527
EPI_ISL_426564
EPI_ISL_421755
EPI_ISL_418155
EPI_ISL_427034
EPI_ISL_426750
EPI_ISL_420145
EPI_ISL_418826
EPI_ISL_426820
EPI_ISL_428253
EPI_ISL_427017
EPI_ISL_417313
EPI_ISL_420930
EPI_ISL_414564
EPI_ISL_420137
EPI_ISL_419960
EPI_ISL_429065
EPI_ISL_417795
EPI_ISL_416491
EPI_ISL_426082

bioRxiv preprint doi: https://doi.org/10.1101/2020.11.06.370999; this version posted November 6, 2020. The copyright holder for this preprint (which was not certified by peer review) is the author/funder, who has granted bioRxiv a license to display the preprint in perpetuity. It is made available under aCC-BY 4.0 International license.

Table S3: GISAID sequence IDs collected on April 25, 2020.

ID
EPI_ISL_420200
EPI_ISL_429561
EPI_ISL_423358
EPI_ISL_423212
EPI_ISL_419218
EPI_ISL_425170
EPI_ISL_419524
EPI_ISL_421683
EPI_ISL_427073
EPI_ISL_415977
EPI_ISL_417638
EPI_ISL_429622
EPI_ISL_423592
EPI_ISL_429607
EPI_ISL_419971
EPI_ISL_424065
EPI_ISL_422938
EPI_ISL_418188
EPI_ISL_418045
EPI_ISL_427324
EPI_ISL_425187
EPI_ISL_426567
EPI_ISL_418712
EPI_ISL_421573
EPI_ISL_426091
EPI_ISL_414438
EPI_ISL_419254
EPI_ISL_430019
EPI_ISL_425609
EPI_ISL_422076
EPI_ISL_419482
EPI_ISL_417854
EPI_ISL_419848
EPI_ISL_429015
EPI_ISL_421729
EPI_ISL_424262
EPI_ISL_416524
EPI_ISL_424008

bioRxiv preprint doi: https://doi.org/10.1101/2020.11.06.370999; this version posted November 6, 2020. The copyright holder for this preprint (which was not certified by peer review) is the author/funder, who has granted bioRxiv a license to display the preprint in perpetuity. It is made available under aCC-BY 4.0 International license.

Table S3: GISAID sequence IDs collected on April 25, 2020.

ID
EPI_ISL_428325
EPI_ISL_421243
EPI_ISL_418720
EPI_ISL_413997
EPI_ISL_417560
EPI_ISL_423044
EPI_ISL_425287
EPI_ISL_427674
EPI_ISL_423122
EPI_ISL_417194
EPI_ISL_422057
EPI_ISL_416721
EPI_ISL_416744
EPI_ISL_429346
EPI_ISL_428347
EPI_ISL_429465
EPI_ISL_428900
EPI_ISL_426074
EPI_ISL_416334
EPI_ISL_423692
EPI_ISL_428800
EPI_ISL_418665
EPI_ISL_425470
EPI_ISL_422746
EPI_ISL_417004
EPI_ISL_419517
EPI_ISL_427531
EPI_ISL_424938
EPI_ISL_423027
EPI_ISL_425784
EPI_ISL_426768
EPI_ISL_424937
EPI_ISL_425511
EPI_ISL_414692
EPI_ISL_428267
EPI_ISL_417426
EPI_ISL_420040
EPI_ISL_418220

bioRxiv preprint doi: https://doi.org/10.1101/2020.11.06.370999; this version posted November 6, 2020. The copyright holder for this preprint (which was not certified by peer review) is the author/funder, who has granted bioRxiv a license to display the preprint in perpetuity. It is made available under aCC-BY 4.0 International license.

Table S3: GISAID sequence IDs collected on April 25, 2020.

ID
EPI_ISL_422164
EPI_ISL_411950
EPI_ISL_420078
EPI_ISL_421743
EPI_ISL_429884
EPI_ISL_422694
EPI_ISL_420326
EPI_ISL_425720
EPI_ISL_419260
EPI_ISL_422557
EPI_ISL_421432
EPI_ISL_422944
EPI_ISL_417675
EPI_ISL_424059
EPI_ISL_418869
EPI_ISL_418882
EPI_ISL_422222
EPI_ISL_424974
EPI_ISL_427545
EPI_ISL_423216
EPI_ISL_418739
EPI_ISL_423856
EPI_ISL_429634
EPI_ISL_421320
EPI_ISL_427455
EPI_ISL_427071
EPI_ISL_429565
EPI_ISL_424857
EPI_ISL_427164
EPI_ISL_422174
EPI_ISL_422115
EPI_ISL_423941
EPI_ISL_422855
EPI_ISL_426059
EPI_ISL_424846
EPI_ISL_418016
EPI_ISL_427106
EPI_ISL_424209

bioRxiv preprint doi: https://doi.org/10.1101/2020.11.06.370999; this version posted November 6, 2020. The copyright holder for this preprint (which was not certified by peer review) is the author/funder, who has granted bioRxiv a license to display the preprint in perpetuity. It is made available under aCC-BY 4.0 International license.

Table S3: GISAID sequence IDs collected on April 25, 2020.

ID
EPI_ISL_417296
EPI_ISL_420570
EPI_ISL_424186
EPI_ISL_426460
EPI_ISL_426653
EPI_ISL_428878
EPI_ISL_420842
EPI_ISL_425455
EPI_ISL_423320
EPI_ISL_420305
EPI_ISL_418768
EPI_ISL_418935
EPI_ISL_426164
EPI_ISL_427692
EPI_ISL_418717
EPI_ISL_425923
EPI_ISL_422317
EPI_ISL_420505
EPI_ISL_416737
EPI_ISL_429583
EPI_ISL_416694
EPI_ISL_425053
EPI_ISL_421430
EPI_ISL_429008
EPI_ISL_423817
EPI_ISL_406970
EPI_ISL_421612
EPI_ISL_415602
EPI_ISL_427127
EPI_ISL_414550
EPI_ISL_417270
EPI_ISL_429558
EPI_ISL_424606
EPI_ISL_429569
EPI_ISL_424521
EPI_ISL_413652
EPI_ISL_423596
EPI_ISL_423663

bioRxiv preprint doi: https://doi.org/10.1101/2020.11.06.370999; this version posted November 6, 2020. The copyright holder for this preprint (which was not certified by peer review) is the author/funder, who has granted bioRxiv a license to display the preprint in perpetuity. It is made available under aCC-BY 4.0 International license.

Table S3: GISAID sequence IDs collected on April 25, 2020.

ID
EPI_ISL_419417
EPI_ISL_419589
EPI_ISL_420117
EPI_ISL_422767
EPI_ISL_426695
EPI_ISL_420010
EPI_ISL_415507
EPI_ISL_421847
EPI_ISL_426683
EPI_ISL_422849
EPI_ISL_426928
EPI_ISL_429631
EPI_ISL_420511
EPI_ISL_429643
EPI_ISL_423639
EPI_ISL_420670
EPI_ISL_421909
EPI_ISL_421453
EPI_ISL_427663
EPI_ISL_420224
EPI_ISL_421311
EPI_ISL_413518
EPI_ISL_421705
EPI_ISL_424041
EPI_ISL_419506
EPI_ISL_414471
EPI_ISL_423693
EPI_ISL_417655
EPI_ISL_422520
EPI_ISL_423195
EPI_ISL_416326
EPI_ISL_425786
EPI_ISL_416584
EPI_ISL_423871
EPI_ISL_414534
EPI_ISL_427063
EPI_ISL_423109
EPI_ISL_420129

bioRxiv preprint doi: https://doi.org/10.1101/2020.11.06.370999; this version posted November 6, 2020. The copyright holder for this preprint (which was not certified by peer review) is the author/funder, who has granted bioRxiv a license to display the preprint in perpetuity. It is made available under aCC-BY 4.0 International license.

Table S3: GISAID sequence IDs collected on April 25, 2020.

ID
EPI_ISL_423484
EPI_ISL_420775
EPI_ISL_429121
EPI_ISL_418337
EPI_ISL_424406
EPI_ISL_419823
EPI_ISL_426624
EPI_ISL_416693
EPI_ISL_420267
EPI_ISL_423507
EPI_ISL_415148
EPI_ISL_414456
EPI_ISL_423406
EPI_ISL_421924
EPI_ISL_422940
EPI_ISL_429119
EPI_ISL_420020
EPI_ISL_419533
EPI_ISL_423584
EPI_ISL_421198
EPI_ISL_420622
EPI_ISL_429397
EPI_ISL_426982
EPI_ISL_419835
EPI_ISL_421984
EPI_ISL_426116
EPI_ISL_428478
EPI_ISL_420393
EPI_ISL_419751
EPI_ISL_424579
EPI_ISL_420053
EPI_ISL_423314
EPI_ISL_421828
EPI_ISL_422504
EPI_ISL_422893
EPI_ISL_416690
EPI_ISL_424963
EPI_ISL_426113

bioRxiv preprint doi: https://doi.org/10.1101/2020.11.06.370999; this version posted November 6, 2020. The copyright holder for this preprint (which was not certified by peer review) is the author/funder, who has granted bioRxiv a license to display the preprint in perpetuity. It is made available under aCC-BY 4.0 International license.

Table S3: GISAID sequence IDs collected on April 25, 2020.

ID
EPI_ISL_421946
EPI_ISL_427484
EPI_ISL_416315
EPI_ISL_422118
EPI_ISL_423030
EPI_ISL_426066
EPI_ISL_421970
EPI_ISL_426918
EPI_ISL_423415
EPI_ISL_421622
EPI_ISL_427448
EPI_ISL_429866
EPI_ISL_426558
EPI_ISL_421368
EPI_ISL_418027
EPI_ISL_416448
EPI_ISL_417260
EPI_ISL_427308
EPI_ISL_421393
EPI_ISL_420009
EPI_ISL_423885
EPI_ISL_420276
EPI_ISL_428927
EPI_ISL_425529
EPI_ISL_420553
EPI_ISL_418925
EPI_ISL_423746
EPI_ISL_422730
EPI_ISL_426985
EPI_ISL_425361
EPI_ISL_427366
EPI_ISL_418232
EPI_ISL_425471
EPI_ISL_421421
EPI_ISL_428288
EPI_ISL_421309
EPI_ISL_416432
EPI_ISL_416758

bioRxiv preprint doi: https://doi.org/10.1101/2020.11.06.370999; this version posted November 6, 2020. The copyright holder for this preprint (which was not certified by peer review) is the author/funder, who has granted bioRxiv a license to display the preprint in perpetuity. It is made available under aCC-BY 4.0 International license.

Table S3: GISAID sequence IDs collected on April 25, 2020.

ID
EPI_ISL_423904
EPI_ISL_426694
EPI_ISL_425241
EPI_ISL_427068
EPI_ISL_424308
EPI_ISL_426506
EPI_ISL_426068
EPI_ISL_423764
EPI_ISL_428352
EPI_ISL_423360
EPI_ISL_421303
EPI_ISL_421390
EPI_ISL_429629
EPI_ISL_426929
EPI_ISL_420214
EPI_ISL_416585
EPI_ISL_419233
EPI_ISL_418981
EPI_ISL_422157
EPI_ISL_425138
EPI_ISL_425301
EPI_ISL_415515
EPI_ISL_421350
EPI_ISL_420630
EPI_ISL_418212
EPI_ISL_429800
EPI_ISL_429290
EPI_ISL_415613
EPI_ISL_415658
EPI_ISL_423497
EPI_ISL_408977
EPI_ISL_417630
EPI_ISL_429624
EPI_ISL_424143
EPI_ISL_420485
EPI_ISL_413020
EPI_ISL_423165
EPI_ISL_425708

bioRxiv preprint doi: https://doi.org/10.1101/2020.11.06.370999; this version posted November 6, 2020. The copyright holder for this preprint (which was not certified by peer review) is the author/funder, who has granted bioRxiv a license to display the preprint in perpetuity. It is made available under aCC-BY 4.0 International license.

Table S3: GISAID sequence IDs collected on April 25, 2020.

ID
EPI_ISL_420496
EPI_ISL_427317
EPI_ISL_423412
EPI_ISL_423075
EPI_ISL_424038
EPI_ISL_416437
EPI_ISL_417133
EPI_ISL_429219
EPI_ISL_429530
EPI_ISL_429737
EPI_ISL_426807
EPI_ISL_425630
EPI_ISL_423211
EPI_ISL_422320
EPI_ISL_424550
EPI_ISL_426955
EPI_ISL_413615
EPI_ISL_423503
EPI_ISL_417973
EPI_ISL_419976
EPI_ISL_421994
EPI_ISL_425982
EPI_ISL_429458
EPI_ISL_425568
EPI_ISL_424864
EPI_ISL_418508
EPI_ISL_417855
EPI_ISL_423126
EPI_ISL_420656
EPI_ISL_423868
EPI_ISL_425600
EPI_ISL_429099
EPI_ISL_418072
EPI_ISL_429038
EPI_ISL_417470
EPI_ISL_423923
EPI_ISL_425133
EPI_ISL_426560

bioRxiv preprint doi: https://doi.org/10.1101/2020.11.06.370999; this version posted November 6, 2020. The copyright holder for this preprint (which was not certified by peer review) is the author/funder, who has granted bioRxiv a license to display the preprint in perpetuity. It is made available under aCC-BY 4.0 International license.

Table S3: GISAID sequence IDs collected on April 25, 2020.

ID
EPI_ISL_429517
EPI_ISL_423879
EPI_ISL_417099
EPI_ISL_420685
EPI_ISL_429599
EPI_ISL_423397
EPI_ISL_417876
EPI_ISL_423667
EPI_ISL_419892
EPI_ISL_429080
EPI_ISL_429079
EPI_ISL_418956
EPI_ISL_420086
EPI_ISL_417472
EPI_ISL_427351
EPI_ISL_419593
EPI_ISL_420935
EPI_ISL_428836
EPI_ISL_417544
EPI_ISL_416594
EPI_ISL_417405
EPI_ISL_425862
EPI_ISL_421829
EPI_ISL_425712
EPI_ISL_427103
EPI_ISL_423716
EPI_ISL_414007
EPI_ISL_419558
EPI_ISL_428960
EPI_ISL_416517
EPI_ISL_423429
EPI_ISL_422697
EPI_ISL_420969
EPI_ISL_426734
EPI_ISL_430093
EPI_ISL_426035
EPI_ISL_420512
EPI_ISL_419587

bioRxiv preprint doi: https://doi.org/10.1101/2020.11.06.370999; this version posted November 6, 2020. The copyright holder for this preprint (which was not certified by peer review) is the author/funder, who has granted bioRxiv a license to display the preprint in perpetuity. It is made available under aCC-BY 4.0 International license.

Table S3: GISAID sequence IDs collected on April 25, 2020.

ID
EPI_ISL_427204
EPI_ISL_417359
EPI_ISL_416581
EPI_ISL_417796
EPI_ISL_415142
EPI_ISL_419684
EPI_ISL_419741
EPI_ISL_416596
EPI_ISL_425401
EPI_ISL_427699
EPI_ISL_422167
EPI_ISL_429299
EPI_ISL_415707
EPI_ISL_423336
EPI_ISL_428670
EPI_ISL_427500
EPI_ISL_425177
EPI_ISL_422603
EPI_ISL_424509
EPI_ISL_419993
EPI_ISL_428709
EPI_ISL_428284
EPI_ISL_421902
EPI_ISL_419943
EPI_ISL_421244
EPI_ISL_419927
EPI_ISL_421720
EPI_ISL_419223
EPI_ISL_418963
EPI_ISL_426289
EPI_ISL_426724
EPI_ISL_424146
EPI_ISL_421776
EPI_ISL_425182
EPI_ISL_414517
EPI_ISL_416443
EPI_ISL_414541
EPI_ISL_422689

bioRxiv preprint doi: https://doi.org/10.1101/2020.11.06.370999; this version posted November 6, 2020. The copyright holder for this preprint (which was not certified by peer review) is the author/funder, who has granted bioRxiv a license to display the preprint in perpetuity. It is made available under aCC-BY 4.0 International license.

Table S3: GISAID sequence IDs collected on April 25, 2020.

ID
EPI_ISL_417742
EPI_ISL_418015
EPI_ISL_421996
EPI_ISL_414643
EPI_ISL_428317
EPI_ISL_422345
EPI_ISL_420521
EPI_ISL_420318
EPI_ISL_424431
EPI_ISL_422265
EPI_ISL_418293
EPI_ISL_422432
EPI_ISL_424197
EPI_ISL_425178
EPI_ISL_420940
EPI_ISL_417432
EPI_ISL_425692
EPI_ISL_417444
EPI_ISL_429821
EPI_ISL_420226
EPI_ISL_414542
EPI_ISL_418025
EPI_ISL_429010
EPI_ISL_425122
EPI_ISL_421301
EPI_ISL_423957
EPI_ISL_423949
EPI_ISL_428870
EPI_ISL_420154
EPI_ISL_422941
EPI_ISL_423246
EPI_ISL_421876
EPI_ISL_430100
EPI_ISL_426075
EPI_ISL_417215
EPI_ISL_419228
EPI_ISL_418777
EPI_ISL_418953

bioRxiv preprint doi: https://doi.org/10.1101/2020.11.06.370999; this version posted November 6, 2020. The copyright holder for this preprint (which was not certified by peer review) is the author/funder, who has granted bioRxiv a license to display the preprint in perpetuity. It is made available under aCC-BY 4.0 International license.

Table S3: GISAID sequence IDs collected on April 25, 2020.

ID
EPI_ISL_418920
EPI_ISL_430020
EPI_ISL_424865
EPI_ISL_423054
EPI_ISL_423175
EPI_ISL_427123
EPI_ISL_425953
EPI_ISL_416499
EPI_ISL_429209
EPI_ISL_427081
EPI_ISL_430001
EPI_ISL_423307
EPI_ISL_417204
EPI_ISL_427629
EPI_ISL_420250
EPI_ISL_423010
EPI_ISL_429603
EPI_ISL_424643
EPI_ISL_408479
EPI_ISL_425941
EPI_ISL_423189
EPI_ISL_425449
EPI_ISL_424874
EPI_ISL_416753
EPI_ISL_418994
EPI_ISL_425944
EPI_ISL_423586
EPI_ISL_413589
EPI_ISL_417575
EPI_ISL_430028
EPI_ISL_423803
EPI_ISL_427129
EPI_ISL_418753
EPI_ISL_429853
EPI_ISL_428395
EPI_ISL_417564
EPI_ISL_420985
EPI_ISL_420382

bioRxiv preprint doi: https://doi.org/10.1101/2020.11.06.370999; this version posted November 6, 2020. The copyright holder for this preprint (which was not certified by peer review) is the author/funder, who has granted bioRxiv a license to display the preprint in perpetuity. It is made available under aCC-BY 4.0 International license.

Table S3: GISAID sequence IDs collected on April 25, 2020.

ID
EPI_ISL_425691
EPI_ISL_427700
EPI_ISL_427065
EPI_ISL_427304
EPI_ISL_416607
EPI_ISL_420321
EPI_ISL_426064
EPI_ISL_421395
EPI_ISL_422253
EPI_ISL_427452
EPI_ISL_423494
EPI_ISL_422422
EPI_ISL_417569
EPI_ISL_418044
EPI_ISL_424128
EPI_ISL_421382
EPI_ISL_422542
EPI_ISL_424176
EPI_ISL_426499
EPI_ISL_424466
EPI_ISL_425358
EPI_ISL_420195
EPI_ISL_424895
EPI_ISL_424414
EPI_ISL_413569
EPI_ISL_424506
EPI_ISL_427566
EPI_ISL_422529
EPI_ISL_403935
EPI_ISL_423637
EPI_ISL_420007
EPI_ISL_412981
EPI_ISL_424397
EPI_ISL_426550
EPI_ISL_417024
EPI_ISL_427385
EPI_ISL_422054
EPI_ISL_415461

bioRxiv preprint doi: https://doi.org/10.1101/2020.11.06.370999; this version posted November 6, 2020. The copyright holder for this preprint (which was not certified by peer review) is the author/funder, who has granted bioRxiv a license to display the preprint in perpetuity. It is made available under aCC-BY 4.0 International license.

Table S3: GISAID sequence IDs collected on April 25, 2020.

ID
EPI_ISL_426937
EPI_ISL_426519
EPI_ISL_427252
EPI_ISL_428334
EPI_ISL_418695
EPI_ISL_428794
EPI_ISL_429183
EPI_ISL_424384
EPI_ISL_427069
EPI_ISL_425167
EPI_ISL_419724
EPI_ISL_416331
EPI_ISL_423961
EPI_ISL_427187
EPI_ISL_424529
EPI_ISL_424440
EPI_ISL_419800
EPI_ISL_425775
EPI_ISL_425992
EPI_ISL_420016
EPI_ISL_416691
EPI_ISL_427175
EPI_ISL_426651
EPI_ISL_428725
EPI_ISL_418351
EPI_ISL_423006
EPI_ISL_423294
EPI_ISL_421507
EPI_ISL_424451
EPI_ISL_429567
EPI_ISL_426956
EPI_ISL_428919
EPI_ISL_419400
EPI_ISL_420993
EPI_ISL_421398
EPI_ISL_429269
EPI_ISL_419663
EPI_ISL_417368

bioRxiv preprint doi: https://doi.org/10.1101/2020.11.06.370999; this version posted November 6, 2020. The copyright holder for this preprint (which was not certified by peer review) is the author/funder, who has granted bioRxiv a license to display the preprint in perpetuity. It is made available under aCC-BY 4.0 International license.

Table S3: GISAID sequence IDs collected on April 25, 2020.

ID
EPI_ISL_428697
EPI_ISL_427191
EPI_ISL_423376
EPI_ISL_428384
EPI_ISL_425789
EPI_ISL_417988
EPI_ISL_422570
EPI_ISL_427022
EPI_ISL_422820
EPI_ISL_414689
EPI_ISL_416033
EPI_ISL_417734
EPI_ISL_425523

Table S4: Clusters identified using a genetic distance threshold within 1% of the distribution of patristic distances within the entire tree. The minimum percentile threshold that maximized the number of clusters was chosen as the optimal threshold by performing multiple clustering runs on randomly sampled patristic distance distributions (1 million for each run) in Phylopart v2 (*46*).

clustername	bootstrap	leafname	branchPath	medianOfDistances	sequencesperCluster
923	1.0	EPI_ISL_426132	6.893599999999978E-4	6.539999999999758E-6	4
923	1.0	EPI_ISL_429638	6.871799999999978E-4	6.539999999999758E-6	4
923	1.0	EPI_ISL_429626	6.893599999999978E-4	6.539999999999758E-6	4
923	1.0	EPI_ISL_429616	6.893599999999978E-4	6.539999999999758E-6	4
1132	1.0	EPI_ISL_418361	6.26129999999999E-4	4.359999999999911E-6	2
1132	1.0	EPI_ISL_425257	6.26129999999999E-4	4.359999999999911E-6	2
1139	1.0	EPI_ISL_424496	6.64909999999999E-4	3.877999999999998E-5	2
1139	1.0	EPI_ISL_422650	6.30489999999999E-4	3.877999999999998E-5	2
1148	1.0	EPI_ISL_419720	6.195899999999992E-4	1.7439999999999643E-5	12
1148	1.0	EPI_ISL_426804	6.28309999999999E-4	1.7439999999999643E-5	12
1148	1.0	EPI_ISL_419989	6.28309999999999E-4	1.7439999999999643E-5	12
1148	1.0	EPI_ISL_427032	6.535599999999993E-4	1.7439999999999643E-5	12
1148	1.0	EPI_ISL_419911	6.28309999999999E-4	1.7439999999999643E-5	12
1148	1.0	EPI_ISL_419983	6.28309999999999E-4	1.7439999999999643E-5	12
1148	1.0	EPI_ISL_419997	6.239499999999991E-4	1.7439999999999643E-5	12
1148	1.0	EPI_ISL_419822	6.28309999999999E-4	1.7439999999999643E-5	12
1148	1.0	EPI_ISL_426807	6.152299999999993E-4	1.7439999999999643E-5	12
1148	1.0	EPI_ISL_419999	6.977599999999991E-4	1.7439999999999643E-5	12
1148	1.0	EPI_ISL_427156	6.566499999999992E-4	1.7439999999999643E-5	12
1148	1.0	EPI_ISL_419852	6.28309999999999E-4	1.7439999999999643E-5	12
1202	1.0	EPI_ISL_414635	6.64899999999999E-4	7.814999999999992E-5	3
1202	1.0	EPI_ISL_418239	7.38689999999999E-4	7.814999999999992E-5	3
1202	1.0	EPI_ISL_414636	7.38689999999999E-4	7.814999999999992E-5	3
1241	1.0	EPI_ISL_426467	6.785999999999987E-4	3.877000000000004E-5	3
1241	1.0	EPI_ISL_426466	6.785999999999987E-4	3.877000000000004E-5	3
1241	1.0	EPI_ISL_426457	7.130099999999987E-4	3.877000000000004E-5	3

bioRxiv preprint doi: https://doi.org/10.1101/2020.11.06.370999; this version posted November 6, 2020. The copyright holder for this preprint (which was not certified by peer review) is the author/funder, who has granted bioRxiv a license to display the preprint in perpetuity. It is made available under aCC-BY 4.0 International license.

Table S4: Clusters identified using a genetic distance threshold within 1% of the distribution of patristic distances within the entire tree. The minimum percentile threshold that maximized the number of clusters was chosen as the optimal threshold by performing multiple clustering runs on randomly sampled patristic distance distributions (1 million for each run) in Phylopart v2 (*46*).

clustername	bootstrap	leafname	branchPath	medianOfDistances	sequencesperCluster
1246	1.0	EPI_ISL_422047	6.785999999999987E-4	4.359999999999911E-6	2
1246	1.0	EPI_ISL_422304	6.785999999999987E-4	4.359999999999911E-6	2
1271	1.0	EPI_ISL_420021	6.501099999999987E-4	4.775999999999977E-5	11
1271	1.0	EPI_ISL_420019	7.089799999999988E-4	4.775999999999977E-5	11
1271	1.0	EPI_ISL_420630	6.501099999999987E-4	4.775999999999977E-5	11
1271	1.0	EPI_ISL_420022	6.845199999999987E-4	4.775999999999977E-5	11
1271	1.0	EPI_ISL_420629	6.501099999999987E-4	4.775999999999977E-5	11
1271	1.0	EPI_ISL_420020	6.501099999999987E-4	4.775999999999977E-5	11
1271	1.0	EPI_ISL_419258	6.479299999999987E-4	4.775999999999977E-5	11
1271	1.0	EPI_ISL_429981	6.738899999999988E-4	4.775999999999977E-5	11
1271	1.0	EPI_ISL_419257	6.804799999999988E-4	4.775999999999977E-5	11
1271	1.0	EPI_ISL_429982	7.487899999999987E-4	4.775999999999977E-5	11
1271	1.0	EPI_ISL_426455	6.501099999999987E-4	4.775999999999977E-5	11
1294	1.0	EPI_ISL_429554	7.036999999999989E-4	7.322999999999987E-5	2
1294	1.0	EPI_ISL_429528	7.03709999999999E-4	7.322999999999987E-5	2
1331	1.0	EPI_ISL_422212	6.26129999999999E-4	4.531999999999995E-5	5
1331	1.0	EPI_ISL_422224	6.67089999999999E-4	4.531999999999995E-5	5
1331	1.0	EPI_ISL_428912	6.62719999999999E-4	4.531999999999995E-5	5
1331	1.0	EPI_ISL_422024	6.67089999999999E-4	4.531999999999995E-5	5
1331	1.0	EPI_ISL_422216	6.64909999999999E-4	4.531999999999995E-5	5
1367	1.0	EPI_ISL_421979	7.601999999999987E-4	6.539999999999758E-6	3
1367	1.0	EPI_ISL_420529	7.623799999999987E-4	6.539999999999758E-6	3
1367	1.0	EPI_ISL_420702	7.623799999999987E-4	6.539999999999758E-6	3
1373	1.0	EPI_ISL_428379	6.451899999999987E-4	3.921999999999997E-5	2
1373	1.0	EPI_ISL_419516	6.800499999999988E-4	3.921999999999997E-5	2
1380	1.0	EPI_ISL_428202	6.69019999999999E-4	7.450999999999985E-5	2

bioRxiv preprint doi: https://doi.org/10.1101/2020.11.06.370999; this version posted November 6, 2020. The copyright holder for this preprint (which was not certified by peer review) is the author/funder, who has granted bioRxiv a license to display the preprint in perpetuity. It is made available under aCC-BY 4.0 International license.

Table S4: Clusters identified using a genetic distance threshold within 1% of the distribution of patristic distances within the entire tree. The minimum percentile threshold that maximized the number of clusters was chosen as the optimal threshold by performing multiple clustering runs on randomly sampled patristic distance distributions (1 million for each run) in Phylopart v2 (*46*).

clustername	bootstrap	leafname	branchPath	medianOfDistances	sequencesperCluster
1380	1.0	EPI_ISL_428205	6.69589999999999E-4	7.450999999999985E-5	2
1436	1.0	EPI_ISL_421391	6.329599999999989E-4	4.098999999999986E-5	4
1436	1.0	EPI_ISL_428385	6.329599999999989E-4	4.098999999999986E-5	4
1436	1.0	EPI_ISL_421623	6.65229999999999E-4	4.098999999999986E-5	4
1436	1.0	EPI_ISL_428394	6.28599999999999E-4	4.098999999999986E-5	4
1443	1.0	EPI_ISL_429588	6.627299999999991E-4	7.318999999999989E-5	2
1443	1.0	EPI_ISL_414625	6.62719999999999E-4	7.318999999999989E-5	2
1483	1.0	EPI_ISL_427742	6.758099999999988E-4	3.877000000000004E-5	2
1483	1.0	EPI_ISL_421636	7.102199999999988E-4	3.877000000000004E-5	2
1507	1.0	EPI_ISL_426837	7.282799999999986E-4	4.317999999999987E-5	10
1507	1.0	EPI_ISL_419943	7.369999999999984E-4	4.317999999999987E-5	10
1507	1.0	EPI_ISL_426860	7.714599999999984E-4	4.317999999999987E-5	10
1507	1.0	EPI_ISL_426729	7.326399999999985E-4	4.317999999999987E-5	10
1507	1.0	EPI_ISL_427764	7.391799999999984E-4	4.317999999999987E-5	10
1507	1.0	EPI_ISL_426862	7.714599999999984E-4	4.317999999999987E-5	10
1507	1.0	EPI_ISL_426725	7.304599999999985E-4	4.317999999999987E-5	10
1507	1.0	EPI_ISL_427682	7.391799999999984E-4	4.317999999999987E-5	10
1507	1.0	EPI_ISL_427775	7.765999999999984E-4	4.317999999999987E-5	10
1507	1.0	EPI_ISL_427683	7.765999999999984E-4	4.317999999999987E-5	10
1531	1.0	EPI_ISL_427095	6.976099999999984E-4	3.877000000000004E-5	4
1531	1.0	EPI_ISL_419956	6.976099999999984E-4	3.877000000000004E-5	4
1531	1.0	EPI_ISL_427094	7.320199999999984E-4	3.877000000000004E-5	4
1531	1.0	EPI_ISL_427092	7.320199999999984E-4	3.877000000000004E-5	4
1601	1.0	EPI_ISL_424170	6.890299999999993E-4	4.359999999999911E-6	2
1601	1.0	EPI_ISL_423020	6.890299999999993E-4	4.359999999999911E-6	2
1605	1.0	EPI_ISL_418204	6.518199999999994E-4	4.133000000000001E-5	6

bioRxiv preprint doi: https://doi.org/10.1101/2020.11.06.370999; this version posted November 6, 2020. The copyright holder for this preprint (which was not certified by peer review) is the author/funder, who has granted bioRxiv a license to display the preprint in perpetuity. It is made available under aCC-BY 4.0 International license.

Table S4: Clusters identified using a genetic distance threshold within 1% of the distribution of patristic distances within the entire tree. The minimum percentile threshold that maximized the number of clusters was chosen as the optimal threshold by performing multiple clustering runs on randomly sampled patristic distance distributions (1 million for each run) in Phylopart v2 (*46*).

clustername	bootstrap	leafname	branchPath	medianOfDistances	sequencesperCluster
1605	1.0	EPI_ISL_429023	7.548399999999995E-4	4.133000000000001E-5	6
1605	1.0	EPI_ISL_417665	6.518199999999994E-4	4.133000000000001E-5	6
1605	1.0	EPI_ISL_426951	6.862499999999994E-4	4.133000000000001E-5	6
1605	1.0	EPI_ISL_430031	6.844299999999994E-4	4.133000000000001E-5	6
1605	1.0	EPI_ISL_418192	6.518199999999994E-4	4.133000000000001E-5	6
1623	1.0	EPI_ISL_430053	6.627499999999991E-4	1.5259999999999796E-5	8
1623	1.0	EPI_ISL_430062	7.368799999999991E-4	1.5259999999999796E-5	8
1623	1.0	EPI_ISL_430056	6.562099999999992E-4	1.5259999999999796E-5	8
1623	1.0	EPI_ISL_420812	6.64929999999999E-4	1.5259999999999796E-5	8
1623	1.0	EPI_ISL_430061	6.64929999999999E-4	1.5259999999999796E-5	8
1623	1.0	EPI_ISL_417977	6.950999999999992E-4	1.5259999999999796E-5	8
1623	1.0	EPI_ISL_430054	6.627499999999991E-4	1.5259999999999796E-5	8
1623	1.0	EPI_ISL_430055	6.605699999999991E-4	1.5259999999999796E-5	8
1640	0.988	EPI_ISL_426748	6.172499999999992E-4	6.539999999999758E-6	4
1640	0.988	EPI_ISL_419952	6.194299999999992E-4	6.539999999999758E-6	4
1640	0.988	EPI_ISL_419954	6.194299999999992E-4	6.539999999999758E-6	4
1640	0.988	EPI_ISL_419880	6.194299999999992E-4	6.539999999999758E-6	4
1666	1.0	EPI_ISL_420759	6.633899999999991E-4	8.686999999999974E-5	49
1666	1.0	EPI_ISL_424161	6.457499999999986E-4	8.686999999999974E-5	49
1666	1.0	EPI_ISL_418076	6.217699999999991E-4	8.686999999999974E-5	49
1666	1.0	EPI_ISL_429128	6.824799999999994E-4	8.686999999999974E-5	49
1666	1.0	EPI_ISL_426746	6.629499999999991E-4	8.686999999999974E-5	49
1666	1.0	EPI_ISL_420704	7.529299999999988E-4	8.686999999999974E-5	49
1666	1.0	EPI_ISL_418690	6.217699999999991E-4	8.686999999999974E-5	49
1666	1.0	EPI_ISL_422035	6.627299999999991E-4	8.686999999999974E-5	49
1666	1.0	EPI_ISL_425525	6.217699999999991E-4	8.686999999999974E-5	49

bioRxiv preprint doi: https://doi.org/10.1101/2020.11.06.370999; this version posted November 6, 2020. The copyright holder for this preprint (which was not certified by peer review) is the author/funder, who has granted bioRxiv a license to display the preprint in perpetuity. It is made available under aCC-BY 4.0 International license.

Table S4: Clusters identified using a genetic distance threshold within 1% of the distribution of patristic distances within the entire tree. The minimum percentile threshold that maximized the number of clusters was chosen as the optimal threshold by performing multiple clustering runs on randomly sampled patristic distance distributions (1 million for each run) in Phylopart v2 (*46*).

clustername	bootstrap	leafname	branchPath	medianOfDistances	sequencesperCluster
1666	1.0	EPI_ISL_420695	6.348499999999989E-4	8.686999999999974E-5	49
1666	1.0	EPI_ISL_429161	6.217699999999991E-4	8.686999999999974E-5	49
1666	1.0	EPI_ISL_423608	6.435699999999987E-4	8.686999999999974E-5	49
1666	1.0	EPI_ISL_425519	6.217699999999991E-4	8.686999999999974E-5	49
1666	1.0	EPI_ISL_423596	6.778099999999989E-4	8.686999999999974E-5	49
1666	1.0	EPI_ISL_422696	6.217699999999991E-4	8.686999999999974E-5	49
1666	1.0	EPI_ISL_429127	6.561799999999992E-4	8.686999999999974E-5	49
1666	1.0	EPI_ISL_422081	7.613999999999992E-4	8.686999999999974E-5	49
1666	1.0	EPI_ISL_423609	6.33229999999999E-4	8.686999999999974E-5	49
1666	1.0	EPI_ISL_421005	7.452199999999988E-4	8.686999999999974E-5	49
1666	1.0	EPI_ISL_420459	6.174099999999992E-4	8.686999999999974E-5	49
1666	1.0	EPI_ISL_425388	7.600699999999985E-4	8.686999999999974E-5	49
1666	1.0	EPI_ISL_423655	6.587699999999987E-4	8.686999999999974E-5	49
1666	1.0	EPI_ISL_424598	6.217699999999991E-4	8.686999999999974E-5	49
1666	1.0	EPI_ISL_419428	6.217699999999991E-4	8.686999999999974E-5	49
1666	1.0	EPI_ISL_423086	6.539999999999992E-4	8.686999999999974E-5	49
1666	1.0	EPI_ISL_422124	7.75279999999999E-4	8.686999999999974E-5	49
1666	1.0	EPI_ISL_422777	6.670799999999989E-4	8.686999999999974E-5	49
1666	1.0	EPI_ISL_423768	6.670799999999989E-4	8.686999999999974E-5	49
1666	1.0	EPI_ISL_423386	6.26129999999999E-4	8.686999999999974E-5	49
1666	1.0	EPI_ISL_422211	7.430399999999988E-4	8.686999999999974E-5	49
1666	1.0	EPI_ISL_422656	7.444199999999989E-4	8.686999999999974E-5	49
1666	1.0	EPI_ISL_422164	7.269899999999992E-4	8.686999999999974E-5	49
1666	1.0	EPI_ISL_429126	6.217699999999991E-4	8.686999999999974E-5	49
1666	1.0	EPI_ISL_429162	6.217699999999991E-4	8.686999999999974E-5	49
1666	1.0	EPI_ISL_424402	6.217699999999991E-4	8.686999999999974E-5	49

bioRxiv preprint doi: https://doi.org/10.1101/2020.11.06.370999; this version posted November 6, 2020. The copyright holder for this preprint (which was not certified by peer review) is the author/funder, who has granted bioRxiv a license to display the preprint in perpetuity. It is made available under aCC-BY 4.0 International license.

Table S4: Clusters identified using a genetic distance threshold within 1% of the distribution of patristic distances within the entire tree. The minimum percentile threshold that maximized the number of clusters was chosen as the optimal threshold by performing multiple clustering runs on randomly sampled patristic distance distributions (1 million for each run) in Phylopart v2 (*46*).

clustername	bootstrap	leafname	branchPath	medianOfDistances	sequencesperCluster
1666	1.0	EPI_ISL_422655	7.444199999999989E-4	8.686999999999974E-5	49
1666	1.0	EPI_ISL_422959	6.130499999999993E-4	8.686999999999974E-5	49
1666	1.0	EPI_ISL_421853	6.195899999999992E-4	8.686999999999974E-5	49
1666	1.0	EPI_ISL_428148	7.12689999999999E-4	8.686999999999974E-5	49
1666	1.0	EPI_ISL_420752	6.802299999999987E-4	8.686999999999974E-5	49
1666	1.0	EPI_ISL_423220	6.26129999999999E-4	8.686999999999974E-5	49
1666	1.0	EPI_ISL_423563	6.37039999999999E-4	8.686999999999974E-5	49
1666	1.0	EPI_ISL_429558	6.736199999999988E-4	8.686999999999974E-5	49
1666	1.0	EPI_ISL_420933	6.842399999999986E-4	8.686999999999974E-5	49
1666	1.0	EPI_ISL_422042	7.452199999999988E-4	8.686999999999974E-5	49
1666	1.0	EPI_ISL_424585	6.217699999999991E-4	8.686999999999974E-5	49
1666	1.0	EPI_ISL_420221	7.185999999999986E-4	8.686999999999974E-5	49
1666	1.0	EPI_ISL_423932	7.151999999999987E-4	8.686999999999974E-5	49
1666	1.0	EPI_ISL_423317	6.217699999999991E-4	8.686999999999974E-5	49
1771	1.0	EPI_ISL_418184	7.875099999999995E-4	3.877999999999998E-5	3
1771	1.0	EPI_ISL_427446	7.530899999999994E-4	3.877999999999998E-5	3
1771	1.0	EPI_ISL_427462	7.530899999999994E-4	3.877999999999998E-5	3
1782	1.0	EPI_ISL_426799	6.759199999999996E-4	4.312999999999995E-5	5
1782	1.0	EPI_ISL_426809	7.125699999999996E-4	4.312999999999995E-5	5
1782	1.0	EPI_ISL_427006	7.083899999999996E-4	4.312999999999995E-5	5
1782	1.0	EPI_ISL_427160	6.802799999999995E-4	4.312999999999995E-5	5
1782	1.0	EPI_ISL_426940	7.146899999999995E-4	4.312999999999995E-5	5
1794	1.0	EPI_ISL_417516	7.125199999999996E-4	7.597000000000007E-5	2
1794	1.0	EPI_ISL_423971	6.409099999999995E-4	7.597000000000007E-5	2
1816	1.0	EPI_ISL_417752	7.471099999999989E-4	6.928999999999941E-5	48
1816	1.0	EPI_ISL_417806	6.602399999999991E-4	6.928999999999941E-5	48

bioRxiv preprint doi: https://doi.org/10.1101/2020.11.06.370999; this version posted November 6, 2020. The copyright holder for this preprint (which was not certified by peer review) is the author/funder, who has granted bioRxiv a license to display the preprint in perpetuity. It is made available under aCC-BY 4.0 International license.

Table S4: Clusters identified using a genetic distance threshold within 1% of the distribution of patristic distances within the entire tree. The minimum percentile threshold that maximized the number of clusters was chosen as the optimal threshold by performing multiple clustering runs on randomly sampled patristic distance distributions (1 million for each run) in Phylopart v2 (*46*).

clustername	bootstrap	leafname	branchPath	medianOfDistances	sequencesperCluster
1816	1.0	EPI_ISL_422914	7.492999999999988E-4	6.928999999999941E-5	48
1816	1.0	EPI_ISL_425409	6.990099999999999E-4	6.928999999999941E-5	48
1816	1.0	EPI_ISL_421501	6.602399999999991E-4	6.928999999999941E-5	48
1816	1.0	EPI_ISL_427721	7.531499999999988E-4	6.928999999999941E-5	48
1816	1.0	EPI_ISL_429729	7.399699999999999E-4	6.928999999999941E-5	48
1816	1.0	EPI_ISL_423443	7.099099999999988E-4	6.928999999999941E-5	48
1816	1.0	EPI_ISL_423797	7.120899999999988E-4	6.928999999999941E-5	48
1816	1.0	EPI_ISL_418206	6.711399999999989E-4	6.928999999999941E-5	48
1816	1.0	EPI_ISL_422568	6.602399999999991E-4	6.928999999999941E-5	48
1816	1.0	EPI_ISL_422954	7.041399999999989E-4	6.928999999999941E-5	48
1816	1.0	EPI_ISL_422858	7.041399999999989E-4	6.928999999999941E-5	48
1816	1.0	EPI_ISL_422856	7.010999999999999E-4	6.928999999999941E-5	48
1816	1.0	EPI_ISL_423445	7.319499999999991E-4	6.928999999999941E-5	48
1816	1.0	EPI_ISL_423163	6.602399999999991E-4	6.928999999999941E-5	48
1816	1.0	EPI_ISL_422620	7.874499999999988E-4	6.928999999999941E-5	48
1816	1.0	EPI_ISL_423643	6.689599999999989E-4	6.928999999999941E-5	48
1816	1.0	EPI_ISL_420791	6.602399999999991E-4	6.928999999999941E-5	48
1816	1.0	EPI_ISL_424042	7.055499999999989E-4	6.928999999999941E-5	48
1816	1.0	EPI_ISL_425322	6.990099999999999E-4	6.928999999999941E-5	48
1816	1.0	EPI_ISL_418207	6.602399999999991E-4	6.928999999999941E-5	48
1816	1.0	EPI_ISL_422658	6.624199999999991E-4	6.928999999999941E-5	48
1816	1.0	EPI_ISL_423603	7.033699999999999E-4	6.928999999999941E-5	48
1816	1.0	EPI_ISL_421560	6.711399999999989E-4	6.928999999999941E-5	48
1816	1.0	EPI_ISL_420048	7.449299999999989E-4	6.928999999999941E-5	48
1816	1.0	EPI_ISL_420058	6.602399999999991E-4	6.928999999999941E-5	48
1816	1.0	EPI_ISL_427136	6.946599999999992E-4	6.928999999999941E-5	48

bioRxiv preprint doi: https://doi.org/10.1101/2020.11.06.370999; this version posted November 6, 2020. The copyright holder for this preprint (which was not certified by peer review) is the author/funder, who has granted bioRxiv a license to display the preprint in perpetuity. It is made available under aCC-BY 4.0 International license.

Table S4: Clusters identified using a genetic distance threshold within 1% of the distribution of patristic distances within the entire tree. The minimum percentile threshold that maximized the number of clusters was chosen as the optimal threshold by performing multiple clustering runs on randomly sampled patristic distance distributions (1 million for each run) in Phylopart v2 (*46*).

clustername	bootstrap	leafname	branchPath	medianOfDistances	sequencesperCluster
1816	1.0	EPI_ISL_422903	7.041399999999989E-4	6.928999999999941E-5	48
1816	1.0	EPI_ISL_418209	6.924699999999992E-4	6.928999999999941E-5	48
1816	1.0	EPI_ISL_422570	7.351199999999992E-4	6.928999999999941E-5	48
1816	1.0	EPI_ISL_420062	7.313399999999992E-4	6.928999999999941E-5	48
1816	1.0	EPI_ISL_423861	7.077299999999989E-4	6.928999999999941E-5	48
1816	1.0	EPI_ISL_423332	7.03369999999999E-4	6.928999999999941E-5	48
1816	1.0	EPI_ISL_425694	6.711399999999989E-4	6.928999999999941E-5	48
1816	1.0	EPI_ISL_429737	7.05559999999999E-4	6.928999999999941E-5	48
1816	1.0	EPI_ISL_423411	7.120899999999988E-4	6.928999999999941E-5	48
1816	1.0	EPI_ISL_423564	7.74479999999999E-4	6.928999999999941E-5	48
1816	1.0	EPI_ISL_420792	6.602399999999991E-4	6.928999999999941E-5	48
1816	1.0	EPI_ISL_424877	6.602399999999991E-4	6.928999999999941E-5	48
1816	1.0	EPI_ISL_422575	6.602399999999991E-4	6.928999999999941E-5	48
1816	1.0	EPI_ISL_418208	6.580599999999992E-4	6.928999999999941E-5	48
1816	1.0	EPI_ISL_418420	6.926199999999992E-4	6.928999999999941E-5	48
1816	1.0	EPI_ISL_423796	7.120899999999988E-4	6.928999999999941E-5	48
1816	1.0	EPI_ISL_423862	6.946499999999991E-4	6.928999999999941E-5	48
1816	1.0	EPI_ISL_422788	6.602399999999991E-4	6.928999999999941E-5	48
1816	1.0	EPI_ISL_417629	6.602399999999991E-4	6.928999999999941E-5	48
1816	1.0	EPI_ISL_422566	6.602399999999991E-4	6.928999999999941E-5	48
1942	1.0	EPI_ISL_427151	6.065099999999994E-4	4.359999999999911E-6	2
1942	1.0	EPI_ISL_427802	6.065099999999994E-4	4.359999999999911E-6	2
1974	1.0	EPI_ISL_426562	7.167099999999987E-4	0.0	6
1974	1.0	EPI_ISL_426563	7.167099999999987E-4	0.0	6
1974	1.0	EPI_ISL_426565	7.167099999999987E-4	0.0	6
1974	1.0	EPI_ISL_426567	7.167099999999987E-4	0.0	6

bioRxiv preprint doi: https://doi.org/10.1101/2020.11.06.370999; this version posted November 6, 2020. The copyright holder for this preprint (which was not certified by peer review) is the author/funder, who has granted bioRxiv a license to display the preprint in perpetuity. It is made available under aCC-BY 4.0 International license.

Table S4: Clusters identified using a genetic distance threshold within 1% of the distribution of patristic distances within the entire tree. The minimum percentile threshold that maximized the number of clusters was chosen as the optimal threshold by performing multiple clustering runs on randomly sampled patristic distance distributions (1 million for each run) in Phylopart v2 (*46*).

clustername	bootstrap	leafname	branchPath	medianOfDistances	sequencesperCluster
1974	1.0	EPI_ISL_426561	7.167099999999987E-4	0.0	6
1974	1.0	EPI_ISL_426566	8.610599999999987E-4	0.0	6
1997	1.0	EPI_ISL_422461	7.510999999999987E-4	7.811999999999988E-5	5
1997	1.0	EPI_ISL_426161	9.322499999999987E-4	7.811999999999988E-5	5
1997	1.0	EPI_ISL_429847	8.248599999999987E-4	7.811999999999988E-5	5
1997	1.0	EPI_ISL_429846	8.248599999999987E-4	7.811999999999988E-5	5
1997	1.0	EPI_ISL_429848	8.248599999999987E-4	7.811999999999988E-5	5
2026	1.0	EPI_ISL_419778	6.627099999999991E-4	4.35999999999911E-6	2
2026	1.0	EPI_ISL_419742	6.627099999999991E-4	4.35999999999911E-6	2
2059	1.0	EPI_ISL_416478	6.539999999999993E-4	7.317999999999995E-5	2
2059	1.0	EPI_ISL_424591	6.539999999999993E-4	7.317999999999995E-5	2
2071	1.0	EPI_ISL_421394	6.130599999999994E-4	4.35999999999911E-6	2
2071	1.0	EPI_ISL_421412	6.130599999999994E-4	4.35999999999911E-6	2
2098	1.0	EPI_ISL_422086	7.704899999999991E-4	3.877999999999998E-5	2
2098	1.0	EPI_ISL_420745	7.36069999999999E-4	3.877999999999998E-5	2
2103	1.0	EPI_ISL_419188	6.26129999999999E-4	7.597000000000007E-5	2
2103	1.0	EPI_ISL_422932	6.977399999999991E-4	7.597000000000007E-5	2
2112	1.0	EPI_ISL_428951	6.976899999999992E-4	8.023999999999987E-5	2
2112	1.0	EPI_ISL_419708	6.218099999999991E-4	8.023999999999987E-5	2
2125	1.0	EPI_ISL_417579	6.67279999999999E-4	4.178999999999988E-5	3
2125	1.0	EPI_ISL_424388	6.67279999999999E-4	4.178999999999988E-5	3
2125	1.0	EPI_ISL_424501	7.003499999999991E-4	4.178999999999988E-5	3
2132	1.0	EPI_ISL_426744	6.217599999999992E-4	3.8979999999999874E-5	2
2132	1.0	EPI_ISL_422532	6.563799999999993E-4	3.8979999999999874E-5	2
2151	1.0	EPI_ISL_423168	6.671299999999991E-4	4.35999999999911E-6	2
2151	1.0	EPI_ISL_422008	6.671299999999991E-4	4.35999999999911E-6	2

bioRxiv preprint doi: https://doi.org/10.1101/2020.11.06.370999; this version posted November 6, 2020. The copyright holder for this preprint (which was not certified by peer review) is the author/funder, who has granted bioRxiv a license to display the preprint in perpetuity. It is made available under aCC-BY 4.0 International license.

Table S4: Clusters identified using a genetic distance threshold within 1% of the distribution of patristic distances within the entire tree. The minimum percentile threshold that maximized the number of clusters was chosen as the optimal threshold by performing multiple clustering runs on randomly sampled patristic distance distributions (1 million for each run) in Phylopart v2 (*46*).

clustername	bootstrap	leafname	branchPath	medianOfDistances	sequencesperCluster
2159	1.0	EPI_ISL_426994	6.65149999999999E-4	5.697999999999988E-5	5
2159	1.0	EPI_ISL_426972	6.627299999999991E-4	5.697999999999988E-5	5
2159	1.0	EPI_ISL_426866	6.26149999999999E-4	5.697999999999988E-5	5
2159	1.0	EPI_ISL_426987	6.80609999999999E-4	5.697999999999988E-5	5
2159	1.0	EPI_ISL_426872	6.30509999999999E-4	5.697999999999988E-5	5
2174	1.0	EPI_ISL_429572	6.949599999999984E-4	8.017999999999979E-5	25
2174	1.0	EPI_ISL_429530	7.40409999999999E-4	8.017999999999979E-5	25
2174	1.0	EPI_ISL_429312	7.184699999999986E-4	8.017999999999979E-5	25
2174	1.0	EPI_ISL_429478	7.228199999999986E-4	8.017999999999979E-5	25
2174	1.0	EPI_ISL_429468	6.949599999999984E-4	8.017999999999979E-5	25
2174	1.0	EPI_ISL_429332	6.753399999999988E-4	8.017999999999979E-5	25
2174	1.0	EPI_ISL_429565	7.075599999999989E-4	8.017999999999979E-5	25
2174	1.0	EPI_ISL_429529	6.949599999999984E-4	8.017999999999979E-5	25
2174	1.0	EPI_ISL_426795	6.66619999999999E-4	8.017999999999979E-5	25
2174	1.0	EPI_ISL_429532	7.831099999999989E-4	8.017999999999979E-5	25
2174	1.0	EPI_ISL_429327	6.949599999999984E-4	8.017999999999979E-5	25
2174	1.0	EPI_ISL_429311	7.075599999999989E-4	8.017999999999979E-5	25
2174	1.0	EPI_ISL_429589	7.119199999999988E-4	8.017999999999979E-5	25
2174	1.0	EPI_ISL_429570	7.271799999999985E-4	8.017999999999979E-5	25
2174	1.0	EPI_ISL_429465	6.949599999999984E-4	8.017999999999979E-5	25
2174	1.0	EPI_ISL_429541	7.838699999999988E-4	8.017999999999979E-5	25
2174	1.0	EPI_ISL_429302	6.949599999999984E-4	8.017999999999979E-5	25
2174	1.0	EPI_ISL_429461	7.621999999999985E-4	8.017999999999979E-5	25
2174	1.0	EPI_ISL_429578	6.949599999999984E-4	8.017999999999979E-5	25
2174	1.0	EPI_ISL_429535	7.228199999999986E-4	8.017999999999979E-5	25
2174	1.0	EPI_ISL_429508	7.40409999999999E-4	8.017999999999979E-5	25

bioRxiv preprint doi: https://doi.org/10.1101/2020.11.06.370999; this version posted November 6, 2020. The copyright holder for this preprint (which was not certified by peer review) is the author/funder, who has granted bioRxiv a license to display the preprint in perpetuity. It is made available under aCC-BY 4.0 International license.

Table S4: Clusters identified using a genetic distance threshold within 1% of the distribution of patristic distances within the entire tree. The minimum percentile threshold that maximized the number of clusters was chosen as the optimal threshold by performing multiple clustering runs on randomly sampled patristic distance distributions (1 million for each run) in Phylopart v2 (*46*).

clustername	bootstrap	leafname	branchPath	medianOfDistances	sequencesperCluster
2174	1.0	EPI_ISL_429566	7.140999999999988E-4	8.017999999999979E-5	25
2174	1.0	EPI_ISL_429310	7.959099999999985E-4	8.017999999999979E-5	25
2174	1.0	EPI_ISL_429294	7.167599999999987E-4	8.017999999999979E-5	25
2174	1.0	EPI_ISL_429300	6.949599999999984E-4	8.017999999999979E-5	25
2229	1.0	EPI_ISL_421382	6.326699999999989E-4	4.0949999999999884E-5	4
2229	1.0	EPI_ISL_429492	6.64899999999999E-4	4.0949999999999884E-5	4
2229	1.0	EPI_ISL_421419	6.348499999999989E-4	4.0949999999999884E-5	4
2229	1.0	EPI_ISL_422538	6.348499999999989E-4	4.0949999999999884E-5	4
2260	1.0	EPI_ISL_418652	7.579799999999987E-4	8.407999999999962E-5	12
2260	1.0	EPI_ISL_418638	7.901899999999988E-4	8.407999999999962E-5	12
2260	1.0	EPI_ISL_418798	7.254499999999986E-4	8.407999999999962E-5	12
2260	1.0	EPI_ISL_418644	7.610599999999986E-4	8.407999999999962E-5	12
2260	1.0	EPI_ISL_420442	7.598599999999986E-4	8.407999999999962E-5	12
2260	1.0	EPI_ISL_427347	7.892799999999988E-4	8.407999999999962E-5	12
2260	1.0	EPI_ISL_421184	7.920899999999987E-4	8.407999999999962E-5	12
2260	1.0	EPI_ISL_417006	7.210899999999987E-4	8.407999999999962E-5	12
2260	1.0	EPI_ISL_424639	7.210899999999987E-4	8.407999999999962E-5	12
2260	1.0	EPI_ISL_427390	7.920899999999987E-4	8.407999999999962E-5	12
2260	1.0	EPI_ISL_427369	8.711599999999986E-4	8.407999999999962E-5	12
2260	1.0	EPI_ISL_424628	7.232699999999986E-4	8.407999999999962E-5	12
2287	1.0	EPI_ISL_429748	7.927999999999987E-4	7.35899999999999E-5	2
2287	1.0	EPI_ISL_429791	7.930299999999987E-4	7.35899999999999E-5	2
2291	1.0	EPI_ISL_429790	7.167299999999988E-4	3.8920000000000014E-5	3
2291	1.0	EPI_ISL_429756	7.534699999999987E-4	3.8920000000000014E-5	3
2291	1.0	EPI_ISL_429767	7.189099999999987E-4	3.8920000000000014E-5	3
2337	0.987	EPI_ISL_430036	6.063499999999995E-4	4.0939999999999943E-5	7

bioRxiv preprint doi: https://doi.org/10.1101/2020.11.06.370999; this version posted November 6, 2020. The copyright holder for this preprint (which was not certified by peer review) is the author/funder, who has granted bioRxiv a license to display the preprint in perpetuity. It is made available under aCC-BY 4.0 International license.

Table S4: Clusters identified using a genetic distance threshold within 1% of the distribution of patristic distances within the entire tree. The minimum percentile threshold that maximized the number of clusters was chosen as the optimal threshold by performing multiple clustering runs on randomly sampled patristic distance distributions (1 million for each run) in Phylopart v2 (*46*).

clustername	bootstrap	leafname	branchPath	medianOfDistances	sequencesperCluster
2337	0.987	EPI_ISL_430023	6.019899999999996E-4	4.093999999999943E-5	7
2337	0.987	EPI_ISL_430029	6.041699999999996E-4	4.093999999999943E-5	7
2337	0.987	EPI_ISL_430027	6.085299999999995E-4	4.093999999999943E-5	7
2337	0.987	EPI_ISL_430034	6.334399999999998E-4	4.093999999999943E-5	7
2337	0.987	EPI_ISL_429121	6.342099999999997E-4	4.093999999999943E-5	7
2337	0.987	EPI_ISL_430020	6.085299999999995E-4	4.093999999999943E-5	7
2369	1.0	EPI_ISL_420575	6.731199999999997E-4	7.969999999999982E-5	13
2369	1.0	EPI_ISL_427162	7.081399999999997E-4	7.969999999999982E-5	13
2369	1.0	EPI_ISL_426053	7.190399999999995E-4	7.969999999999982E-5	13
2369	1.0	EPI_ISL_426839	7.485999999999997E-4	7.969999999999982E-5	13
2369	1.0	EPI_ISL_421578	6.517999999999994E-4	7.969999999999982E-5	13
2369	1.0	EPI_ISL_419703	6.774799999999996E-4	7.969999999999982E-5	13
2369	1.0	EPI_ISL_424304	6.796599999999996E-4	7.969999999999982E-5	13
2369	1.0	EPI_ISL_421363	6.840199999999995E-4	7.969999999999982E-5	13
2369	1.0	EPI_ISL_427562	6.539799999999993E-4	7.969999999999982E-5	13
2369	1.0	EPI_ISL_427633	6.539799999999993E-4	7.969999999999982E-5	13
2369	1.0	EPI_ISL_428388	6.696099999999997E-4	7.969999999999982E-5	13
2369	1.0	EPI_ISL_420296	6.731199999999997E-4	7.969999999999982E-5	13
2369	1.0	EPI_ISL_421720	6.539799999999993E-4	7.969999999999982E-5	13
2413	0.987	EPI_ISL_426159	6.708799999999997E-4	3.875999999999988E-5	3
2413	0.987	EPI_ISL_422463	6.364799999999996E-4	3.875999999999988E-5	3
2413	0.987	EPI_ISL_419651	6.364799999999996E-4	3.875999999999988E-5	3
2422	1.0	EPI_ISL_416509	6.321799999999998E-4	4.093999999999943E-5	3
2422	1.0	EPI_ISL_416512	5.999599999999996E-4	4.093999999999943E-5	3
2422	1.0	EPI_ISL_416513	6.343599999999997E-4	4.093999999999943E-5	3
2434	1.0	EPI_ISL_429639	6.847999999999994E-4	8.03599999999998E-5	22

bioRxiv preprint doi: https://doi.org/10.1101/2020.11.06.370999; this version posted November 6, 2020. The copyright holder for this preprint (which was not certified by peer review) is the author/funder, who has granted bioRxiv a license to display the preprint in perpetuity. It is made available under aCC-BY 4.0 International license.

Table S4: Clusters identified using a genetic distance threshold within 1% of the distribution of patristic distances within the entire tree. The minimum percentile threshold that maximized the number of clusters was chosen as the optimal threshold by performing multiple clustering runs on randomly sampled patristic distance distributions (1 million for each run) in Phylopart v2 (*46*).

clustername	bootstrap	leafname	branchPath	medianOfDistances	sequencesperCluster
2434	1.0	EPI_ISL_426118	6.30479999999999E-4	8.03599999999998E-5	22
2434	1.0	EPI_ISL_429644	7.11729999999996E-4	8.03599999999998E-5	22
2434	1.0	EPI_ISL_429605	6.38829999999996E-4	8.03599999999998E-5	22
2434	1.0	EPI_ISL_427198	6.62699999999991E-4	8.03599999999998E-5	22
2434	1.0	EPI_ISL_427248	6.28299999999991E-4	8.03599999999998E-5	22
2434	1.0	EPI_ISL_427235	6.17399999999993E-4	8.03599999999998E-5	22
2434	1.0	EPI_ISL_427211	6.28299999999991E-4	8.03599999999998E-5	22
2434	1.0	EPI_ISL_424172	6.93399999999992E-4	8.03599999999998E-5	22
2434	1.0	EPI_ISL_424293	6.28299999999991E-4	8.03599999999998E-5	22
2434	1.0	EPI_ISL_424237	6.28299999999991E-4	8.03599999999998E-5	22
2434	1.0	EPI_ISL_427268	6.71559999999996E-4	8.03599999999998E-5	22
2434	1.0	EPI_ISL_424299	7.18879999999994E-4	8.03599999999998E-5	22
2434	1.0	EPI_ISL_429633	6.33629999999997E-4	8.03599999999998E-5	22
2434	1.0	EPI_ISL_426105	6.08679999999995E-4	8.03599999999998E-5	22
2434	1.0	EPI_ISL_426090	6.40899999999996E-4	8.03599999999998E-5	22
2434	1.0	EPI_ISL_424300	6.28299999999991E-4	8.03599999999998E-5	22
2434	1.0	EPI_ISL_424325	6.58339999999992E-4	8.03599999999998E-5	22
2434	1.0	EPI_ISL_429629	7.46139999999996E-4	8.03599999999998E-5	22
2434	1.0	EPI_ISL_424187	6.19579999999992E-4	8.03599999999998E-5	22
2434	1.0	EPI_ISL_424290	6.15219999999993E-4	8.03599999999998E-5	22
2434	1.0	EPI_ISL_424291	6.30479999999999E-4	8.03599999999998E-5	22
2483	1.0	EPI_ISL_423021	6.64919999999997E-4	4.35999999999911E-6	4
2483	1.0	EPI_ISL_423019	6.64919999999997E-4	4.35999999999911E-6	4
2483	1.0	EPI_ISL_424234	6.64919999999997E-4	4.35999999999911E-6	4
2483	1.0	EPI_ISL_424239	6.64919999999997E-4	4.35999999999911E-6	4
2495	1.0	EPI_ISL_429494	6.49889999999994E-4	8.24899999999973E-5	50

bioRxiv preprint doi: https://doi.org/10.1101/2020.11.06.370999; this version posted November 6, 2020. The copyright holder for this preprint (which was not certified by peer review) is the author/funder, who has granted bioRxiv a license to display the preprint in perpetuity. It is made available under aCC-BY 4.0 International license.

Table S4: Clusters identified using a genetic distance threshold within 1% of the distribution of patristic distances within the entire tree. The minimum percentile threshold that maximized the number of clusters was chosen as the optimal threshold by performing multiple clustering runs on randomly sampled patristic distance distributions (1 million for each run) in Phylopart v2 (*46*).

clustername	bootstrap	leafname	branchPath	medianOfDistances	sequencesperCluster
2495	1.0	EPI_ISL_429531	6.526499999999994E-4	8.248999999999973E-5	50
2495	1.0	EPI_ISL_429540	6.086799999999995E-4	8.248999999999973E-5	50
2495	1.0	EPI_ISL_429493	6.408999999999996E-4	8.248999999999973E-5	50
2495	1.0	EPI_ISL_429563	6.086799999999995E-4	8.248999999999973E-5	50
2495	1.0	EPI_ISL_429130	6.430799999999995E-4	8.248999999999973E-5	50
2495	1.0	EPI_ISL_429321	7.140599999999997E-4	8.248999999999973E-5	50
2495	1.0	EPI_ISL_429527	6.868099999999994E-4	8.248999999999973E-5	50
2495	1.0	EPI_ISL_429463	6.796599999999996E-4	8.248999999999973E-5	50
2495	1.0	EPI_ISL_429467	6.938299999999993E-4	8.248999999999973E-5	50
2495	1.0	EPI_ISL_429322	6.086799999999995E-4	8.248999999999973E-5	50
2495	1.0	EPI_ISL_417682	6.780899999999996E-4	8.248999999999973E-5	50
2495	1.0	EPI_ISL_429336	6.496199999999994E-4	8.248999999999973E-5	50
2495	1.0	EPI_ISL_429307	6.086799999999995E-4	8.248999999999973E-5	50
2495	1.0	EPI_ISL_429552	6.086799999999995E-4	8.248999999999973E-5	50
2495	1.0	EPI_ISL_429506	6.086799999999995E-4	8.248999999999973E-5	50
2495	1.0	EPI_ISL_429132	7.051899999999997E-4	8.248999999999973E-5	50
2495	1.0	EPI_ISL_429293	6.824499999999995E-4	8.248999999999973E-5	50
2495	1.0	EPI_ISL_429477	6.086799999999995E-4	8.248999999999973E-5	50
2495	1.0	EPI_ISL_429134	7.182599999999994E-4	8.248999999999973E-5	50
2495	1.0	EPI_ISL_424622	6.889899999999994E-4	8.248999999999973E-5	50
2495	1.0	EPI_ISL_429297	6.796599999999996E-4	8.248999999999973E-5	50
2495	1.0	EPI_ISL_429519	6.086799999999995E-4	8.248999999999973E-5	50
2495	1.0	EPI_ISL_429521	6.387099999999996E-4	8.248999999999973E-5	50
2495	1.0	EPI_ISL_429285	6.517999999999994E-4	8.248999999999973E-5	50
2495	1.0	EPI_ISL_429488	6.086799999999995E-4	8.248999999999973E-5	50
2495	1.0	EPI_ISL_429304	6.824499999999995E-4	8.248999999999973E-5	50

bioRxiv preprint doi: https://doi.org/10.1101/2020.11.06.370999; this version posted November 6, 2020. The copyright holder for this preprint (which was not certified by peer review) is the author/funder, who has granted bioRxiv a license to display the preprint in perpetuity. It is made available under aCC-BY 4.0 International license.

Table S4: Clusters identified using a genetic distance threshold within 1% of the distribution of patristic distances within the entire tree. The minimum percentile threshold that maximized the number of clusters was chosen as the optimal threshold by performing multiple clustering runs on randomly sampled patristic distance distributions (1 million for each run) in Phylopart v2 (*46*).

clustername	bootstrap	leafname	branchPath	medianOfDistances	sequencesperCluster
2495	1.0	EPI_ISL_429574	6.086799999999995E-4	8.248999999999973E-5	50
2495	1.0	EPI_ISL_429331	6.452599999999995E-4	8.248999999999973E-5	50
2495	1.0	EPI_ISL_429546	6.539799999999993E-4	8.248999999999973E-5	50
2495	1.0	EPI_ISL_429305	6.086799999999995E-4	8.248999999999973E-5	50
2495	1.0	EPI_ISL_429286	6.173999999999993E-4	8.248999999999973E-5	50
2495	1.0	EPI_ISL_429557	6.086799999999995E-4	8.248999999999973E-5	50
2495	1.0	EPI_ISL_429472	6.086799999999995E-4	8.248999999999973E-5	50
2495	1.0	EPI_ISL_429318	6.086799999999995E-4	8.248999999999973E-5	50
2495	1.0	EPI_ISL_429489	6.299999999999998E-4	8.248999999999973E-5	50
2495	1.0	EPI_ISL_429479	6.343599999999997E-4	8.248999999999973E-5	50
2495	1.0	EPI_ISL_429483	6.086799999999995E-4	8.248999999999973E-5	50
2495	1.0	EPI_ISL_415648	6.496199999999994E-4	8.248999999999973E-5	50
2495	1.0	EPI_ISL_424532	6.780899999999996E-4	8.248999999999973E-5	50
2495	1.0	EPI_ISL_429556	6.539799999999993E-4	8.248999999999973E-5	50
2495	1.0	EPI_ISL_429562	6.086799999999995E-4	8.248999999999973E-5	50
2495	1.0	EPI_ISL_429543	6.086799999999995E-4	8.248999999999973E-5	50
2495	1.0	EPI_ISL_429496	6.086799999999995E-4	8.248999999999973E-5	50
2495	1.0	EPI_ISL_429289	6.477299999999995E-4	8.248999999999973E-5	50
2495	1.0	EPI_ISL_429485	6.086799999999995E-4	8.248999999999973E-5	50
2495	1.0	EPI_ISL_429481	6.086799999999995E-4	8.248999999999973E-5	50
2495	1.0	EPI_ISL_417690	7.613899999999994E-4	8.248999999999973E-5	50
2495	1.0	EPI_ISL_424400	6.780899999999996E-4	8.248999999999973E-5	50
2495	1.0	EPI_ISL_429131	6.086799999999995E-4	8.248999999999973E-5	50
2597	1.0	EPI_ISL_425263	6.387299999999996E-4	3.874999999999994E-5	3
2597	1.0	EPI_ISL_423384	6.043399999999995E-4	3.874999999999994E-5	3
2597	1.0	EPI_ISL_423383	6.021599999999996E-4	3.874999999999994E-5	3

bioRxiv preprint doi: https://doi.org/10.1101/2020.11.06.370999; this version posted November 6, 2020. The copyright holder for this preprint (which was not certified by peer review) is the author/funder, who has granted bioRxiv a license to display the preprint in perpetuity. It is made available under aCC-BY 4.0 International license.

bioRxiv preprint doi: https://doi.org/10.1101/2020.11.06.370999; this version posted November 6, 2020. The copyright holder for this preprint (which was not certified by peer review) is the author/funder, who has granted bioRxiv a license to display the preprint in perpetuity. It is made available under aCC-BY 4.0 International license.

Table S4: Clusters identified using a genetic distance threshold within 1% of the distribution of patristic distances within the entire tree. The minimum percentile threshold that maximized the number of clusters was chosen as the optimal threshold by performing multiple clustering runs on randomly sampled patristic distance distributions (1 million for each run) in Phylopart v2 (*46*).

clustername	bootstrap	leafname	branchPath	medianOfDistances	sequencesperCluster
2602	1.0	EPI_ISL_429656	6.021299999999995E-4	3.874999999999994E-5	2
2602	1.0	EPI_ISL_422609	6.365199999999996E-4	3.874999999999994E-5	2
2607	1.0	EPI_ISL_428892	6.343399999999996E-4	3.874999999999994E-5	3
2607	1.0	EPI_ISL_428902	6.343399999999996E-4	3.874999999999994E-5	3
2607	1.0	EPI_ISL_420579	5.999499999999996E-4	3.874999999999994E-5	3
2616	1.0	EPI_ISL_424311	6.759399999999995E-4	7.598999999999996E-5	2
2616	1.0	EPI_ISL_425912	6.043099999999995E-4	7.598999999999996E-5	2
2645	1.0	EPI_ISL_429569	6.002499999999997E-4	3.874999999999994E-5	2
2645	1.0	EPI_ISL_429464	6.346399999999997E-4	3.874999999999994E-5	2
2690	1.0	EPI_ISL_421503	6.799199999999995E-4	4.359999999999911E-6	2
2690	1.0	EPI_ISL_420054	6.799199999999995E-4	4.359999999999911E-6	2
2693	1.0	EPI_ISL_421300	6.771299999999995E-4	3.874999999999994E-5	2
2693	1.0	EPI_ISL_416523	6.427399999999995E-4	3.874999999999994E-5	2
2696	1.0	EPI_ISL_426884	6.771399999999996E-4	4.6029999999999856E-5	7
2696	1.0	EPI_ISL_422636	6.735099999999997E-4	4.6029999999999856E-5	7
2696	1.0	EPI_ISL_426887	7.121699999999996E-4	4.6029999999999856E-5	7
2696	1.0	EPI_ISL_425760	6.449199999999995E-4	4.6029999999999856E-5	7
2696	1.0	EPI_ISL_425692	6.449199999999995E-4	4.6029999999999856E-5	7
2696	1.0	EPI_ISL_417830	6.449199999999995E-4	4.6029999999999856E-5	7
2696	1.0	EPI_ISL_428923	6.793099999999995E-4	4.6029999999999856E-5	7
2709	1.0	EPI_ISL_426668	6.733899999999996E-4	3.841999999999995E-5	2
2709	1.0	EPI_ISL_426654	7.074499999999997E-4	3.841999999999995E-5	2
2714	1.0	EPI_ISL_419915	7.482599999999996E-4	4.359999999999911E-6	2
2714	1.0	EPI_ISL_426768	7.482599999999996E-4	4.359999999999911E-6	2
2722	1.0	EPI_ISL_425992	6.408899999999995E-4	0.0	5
2722	1.0	EPI_ISL_425906	6.408899999999995E-4	0.0	5

Table S4: Clusters identified using a genetic distance threshold within 1% of the distribution of patristic distances within the entire tree. The minimum percentile threshold that maximized the number of clusters was chosen as the optimal threshold by performing multiple clustering runs on randomly sampled patristic distance distributions (1 million for each run) in Phylopart v2 (*46*).

clustername	bootstrap	leafname	branchPath	medianOfDistances	sequencesperCluster
2722	1.0	EPI_ISL_425907	6.408899999999995E-4	0.0	5
2722	1.0	EPI_ISL_425866	6.752999999999996E-4	0.0	5
2722	1.0	EPI_ISL_426003	6.408899999999995E-4	0.0	5
2733	1.0	EPI_ISL_414630	6.818199999999994E-4	7.594999999999998E-5	3
2733	1.0	EPI_ISL_428360	6.796399999999995E-4	7.594999999999998E-5	3
2733	1.0	EPI_ISL_418237	7.534099999999995E-4	7.594999999999998E-5	3
2747	1.0	EPI_ISL_428355	6.818299999999995E-4	0.0	5
2747	1.0	EPI_ISL_428351	6.474299999999994E-4	0.0	5
2747	1.0	EPI_ISL_428356	6.474299999999994E-4	0.0	5
2747	1.0	EPI_ISL_416498	6.474299999999994E-4	0.0	5
2747	1.0	EPI_ISL_428357	6.474299999999994E-4	0.0	5
2772	1.0	EPI_ISL_421332	5.999499999999996E-4	4.0939999999999943E-5	15
2772	1.0	EPI_ISL_423011	6.321699999999997E-4	4.0939999999999943E-5	15
2772	1.0	EPI_ISL_425150	6.321699999999997E-4	4.0939999999999943E-5	15
2772	1.0	EPI_ISL_425149	5.999499999999996E-4	4.0939999999999943E-5	15
2772	1.0	EPI_ISL_421302	6.321699999999997E-4	4.0939999999999943E-5	15
2772	1.0	EPI_ISL_421287	5.999499999999996E-4	4.0939999999999943E-5	15
2772	1.0	EPI_ISL_427529	5.999499999999996E-4	4.0939999999999943E-5	15
2772	1.0	EPI_ISL_424945	6.259299999999998E-4	4.0939999999999943E-5	15
2772	1.0	EPI_ISL_425159	7.037699999999997E-4	4.0939999999999943E-5	15
2772	1.0	EPI_ISL_426092	6.278999999999998E-4	4.0939999999999943E-5	15
2772	1.0	EPI_ISL_416642	5.999499999999996E-4	4.0939999999999943E-5	15
2772	1.0	EPI_ISL_416491	5.999499999999996E-4	4.0939999999999943E-5	15
2772	1.0	EPI_ISL_416492	5.999499999999996E-4	4.0939999999999943E-5	15
2772	1.0	EPI_ISL_421311	6.321699999999997E-4	4.0939999999999943E-5	15
2772	1.0	EPI_ISL_422994	5.999499999999996E-4	4.0939999999999943E-5	15

bioRxiv preprint doi: https://doi.org/10.1101/2020.11.06.370999; this version posted November 6, 2020. The copyright holder for this preprint (which was not certified by peer review) is the author/funder, who has granted bioRxiv a license to display the preprint in perpetuity. It is made available under aCC-BY 4.0 International license.

Table S4: Clusters identified using a genetic distance threshold within 1% of the distribution of patristic distances within the entire tree. The minimum percentile threshold that maximized the number of clusters was chosen as the optimal threshold by performing multiple clustering runs on randomly sampled patristic distance distributions (1 million for each run) in Phylopart v2 (*46*).

clustername	bootstrap	leafname	branchPath	medianOfDistances	sequencesperCluster
2819	1.0	EPI_ISL_420573	6.321799999999998E-4	7.969999999999982E-5	10
2819	1.0	EPI_ISL_427592	5.955999999999997E-4	7.969999999999982E-5	10
2819	1.0	EPI_ISL_429129	7.015899999999999E-4	7.969999999999982E-5	10
2819	1.0	EPI_ISL_419702	6.321799999999998E-4	7.969999999999982E-5	10
2819	1.0	EPI_ISL_421728	6.665799999999998E-4	7.969999999999982E-5	10
2819	1.0	EPI_ISL_428772	6.256399999999999E-4	7.969999999999982E-5	10
2819	1.0	EPI_ISL_421675	6.321799999999998E-4	7.969999999999982E-5	10
2819	1.0	EPI_ISL_418041	5.955999999999997E-4	7.969999999999982E-5	10
2819	1.0	EPI_ISL_427607	6.299999999999998E-4	7.969999999999982E-5	10
2819	1.0	EPI_ISL_426128	6.607399999999999E-4	7.969999999999982E-5	10
2846	1.0	EPI_ISL_427491	5.868699999999999E-4	3.874999999999994E-5	2
2846	1.0	EPI_ISL_429641	6.212599999999999E-4	3.874999999999994E-5	2
2861	1.0	EPI_ISL_419864	6.628199999999998E-4	3.874999999999994E-5	3
2861	1.0	EPI_ISL_426683	6.993899999999998E-4	3.874999999999994E-5	3
2861	1.0	EPI_ISL_419844	6.649999999999998E-4	3.874999999999994E-5	3
2878	1.0	EPI_ISL_424215	6.621999999999998E-4	7.531999999999981E-5	6
2878	1.0	EPI_ISL_426100	6.321699999999997E-4	7.531999999999981E-5	6
2878	1.0	EPI_ISL_424294	7.015899999999998E-4	7.531999999999981E-5	6
2878	1.0	EPI_ISL_424166	6.709199999999998E-4	7.531999999999981E-5	6
2878	1.0	EPI_ISL_427212	7.031299999999997E-4	7.531999999999981E-5	6
2878	1.0	EPI_ISL_424263	6.321699999999997E-4	7.531999999999981E-5	6
2901	1.0	EPI_ISL_420056	6.190099999999997E-4	7.592999999999988E-5	2
2901	1.0	EPI_ISL_421509	6.905799999999998E-4	7.592999999999988E-5	2
2960	1.0	EPI_ISL_425643	6.598699999999999E-4	4.359999999999911E-6	2
2960	1.0	EPI_ISL_428365	6.598699999999999E-4	4.359999999999911E-6	2
2980	1.0	EPI_ISL_422419	6.661099999999997E-4	3.874999999999994E-5	2

bioRxiv preprint doi: https://doi.org/10.1101/2020.11.06.370999; this version posted November 6, 2020. The copyright holder for this preprint (which was not certified by peer review) is the author/funder, who has granted bioRxiv a license to display the preprint in perpetuity. It is made available under aCC-BY 4.0 International license.

Table S4: Clusters identified using a genetic distance threshold within 1% of the distribution of patristic distances within the entire tree. The minimum percentile threshold that maximized the number of clusters was chosen as the optimal threshold by performing multiple clustering runs on randomly sampled patristic distance distributions (1 million for each run) in Phylopart v2 (*46*).

clustername	bootstrap	leafname	branchPath	medianOfDistances	sequencesperCluster
2980	1.0	EPI_ISL_426632	6.317199999999997E-4	3.874999999999994E-5	2
3010	1.0	EPI_ISL_420150	6.299599999999997E-4	4.359999999999911E-6	4
3010	1.0	EPI_ISL_429262	6.299599999999997E-4	4.359999999999911E-6	4
3010	1.0	EPI_ISL_429551	6.299599999999997E-4	4.359999999999911E-6	4
3010	1.0	EPI_ISL_425758	6.299599999999997E-4	4.359999999999911E-6	4
3018	1.0	EPI_ISL_415477	6.670099999999997E-4	7.361E-5	2
3018	1.0	EPI_ISL_415525	6.665399999999996E-4	7.361E-5	2
3042	1.0	EPI_ISL_421378	6.430599999999994E-4	8.028999999999979E-5	11
3042	1.0	EPI_ISL_421635	6.430599999999994E-4	8.028999999999979E-5	11
3042	1.0	EPI_ISL_422546	7.483299999999995E-4	8.028999999999979E-5	11
3042	1.0	EPI_ISL_426317	6.430599999999994E-4	8.028999999999979E-5	11
3042	1.0	EPI_ISL_420588	6.430599999999994E-4	8.028999999999979E-5	11
3042	1.0	EPI_ISL_421630	7.074999999999997E-4	8.028999999999979E-5	11
3042	1.0	EPI_ISL_427547	6.430599999999994E-4	8.028999999999979E-5	11
3042	1.0	EPI_ISL_424931	7.102699999999996E-4	8.028999999999979E-5	11
3042	1.0	EPI_ISL_422519	6.752799999999996E-4	8.028999999999979E-5	11
3042	1.0	EPI_ISL_422534	6.752799999999996E-4	8.028999999999979E-5	11
3042	1.0	EPI_ISL_424930	7.102699999999996E-4	8.028999999999979E-5	11
3076	1.0	EPI_ISL_418964	7.395399999999997E-4	4.359999999999911E-6	2
3076	1.0	EPI_ISL_418963	7.395399999999997E-4	4.359999999999911E-6	2
3079	1.0	EPI_ISL_427799	5.934099999999997E-4	3.874999999999994E-5	3
3079	1.0	EPI_ISL_420238	6.277999999999998E-4	3.874999999999994E-5	3
3079	1.0	EPI_ISL_427795	5.934099999999997E-4	3.874999999999994E-5	3
3089	1.0	EPI_ISL_420301	6.212599999999999E-4	3.874999999999994E-5	2
3089	1.0	EPI_ISL_421721	5.868699999999999E-4	3.874999999999994E-5	2
3093	1.0	EPI_ISL_424306	5.846899999999999E-4	4.359999999999911E-6	2

bioRxiv preprint doi: https://doi.org/10.1101/2020.11.06.370999; this version posted November 6, 2020. The copyright holder for this preprint (which was not certified by peer review) is the author/funder, who has granted bioRxiv a license to display the preprint in perpetuity. It is made available under aCC-BY 4.0 International license.

bioRxiv preprint doi: https://doi.org/10.1101/2020.11.06.370999; this version posted November 6, 2020. The copyright holder for this preprint (which was not certified by peer review) is the author/funder, who has granted bioRxiv a license to display the preprint in perpetuity. It is made available under aCC-BY 4.0 International license.

Table S4: Clusters identified using a genetic distance threshold within 1% of the distribution of patristic distances within the entire tree. The minimum percentile threshold that maximized the number of clusters was chosen as the optimal threshold by performing multiple clustering runs on randomly sampled patristic distance distributions (1 million for each run) in Phylopart v2 (*46*).

clustername	bootstrap	leafname	branchPath	medianOfDistances	sequencesperCluster
3093	1.0	EPI_ISL_424339	5.846899999999999E-4	4.359999999999911E-6	2
3103	1.0	EPI_ISL_415649	6.299699999999999E-4	4.121999999999998E-5	3
3103	1.0	EPI_ISL_413572	5.955899999999998E-4	4.121999999999998E-5	3
3103	1.0	EPI_ISL_420322	6.280899999999999E-4	4.121999999999998E-5	3
3111	1.0	EPI_ISL_426028	6.672299999999998E-4	7.600000000000011E-5	2
3111	1.0	EPI_ISL_417707	5.955899999999997E-4	7.600000000000011E-5	2
3114	1.0	EPI_ISL_429482	6.302799999999998E-4	7.532000000000003E-5	3
3114	1.0	EPI_ISL_429480	6.324499999999997E-4	7.532000000000003E-5	3
3114	1.0	EPI_ISL_429581	6.324399999999997E-4	7.532000000000003E-5	3
3123	1.0	EPI_ISL_427476	5.890499999999998E-4	3.874999999999994E-5	3
3123	1.0	EPI_ISL_418974	6.234399999999998E-4	3.874999999999994E-5	3
3123	1.0	EPI_ISL_418200	5.868699999999999E-4	3.874999999999994E-5	3
3136	1.0	EPI_ISL_426124	5.890499999999998E-4	3.876999999999982E-5	2
3136	1.0	EPI_ISL_427554	6.234599999999998E-4	3.876999999999982E-5	2
3140	1.0	EPI_ISL_426979	7.355999999999998E-4	4.359999999999911E-6	2
3140	1.0	EPI_ISL_429013	7.355999999999998E-4	4.359999999999911E-6	2
3145	1.0	EPI_ISL_426086	6.266799999999998E-4	3.951999999999992E-5	3
3145	1.0	EPI_ISL_415481	5.915199999999998E-4	3.951999999999992E-5	3
3145	1.0	EPI_ISL_424330	5.915199999999998E-4	3.951999999999992E-5	3
3198	1.0	EPI_ISL_421316	6.913899999999992E-4	7.592999999999988E-5	2
3198	1.0	EPI_ISL_421312	7.629599999999993E-4	7.592999999999988E-5	2
3201	1.0	EPI_ISL_426536	6.520399999999992E-4	3.874999999999994E-5	2
3201	1.0	EPI_ISL_426526	6.864299999999993E-4	3.874999999999994E-5	2
3208	1.0	EPI_ISL_418893	6.755299999999995E-4	8.029999999999995E-5	7
3208	1.0	EPI_ISL_417376	6.520399999999992E-4	8.029999999999995E-5	7
3208	1.0	EPI_ISL_423028	6.777099999999994E-4	8.029999999999995E-5	7

Table S4: Clusters identified using a genetic distance threshold within 1% of the distribution of patristic distances within the entire tree. The minimum percentile threshold that maximized the number of clusters was chosen as the optimal threshold by performing multiple clustering runs on randomly sampled patristic distance distributions (1 million for each run) in Phylopart v2 (*46*).

clustername	bootstrap	leafname	branchPath	medianOfDistances	sequencesperCluster
3208	1.0	EPI_ISL_426627	7.550899999999994E-4	8.029999999999995E-5	7
3208	1.0	EPI_ISL_418036	6.498599999999993E-4	8.029999999999995E-5	7
3208	1.0	EPI_ISL_428335	6.520399999999992E-4	8.029999999999995E-5	7
3208	1.0	EPI_ISL_424868	7.170799999999994E-4	8.029999999999995E-5	7
3222	1.0	EPI_ISL_420814	6.476799999999993E-4	4.341999999999996E-5	6
3222	1.0	EPI_ISL_430048	6.411399999999995E-4	4.341999999999996E-5	6
3222	1.0	EPI_ISL_420815	6.801999999999994E-4	4.341999999999996E-5	6
3222	1.0	EPI_ISL_420824	6.801999999999994E-4	4.341999999999996E-5	6
3222	1.0	EPI_ISL_421563	6.801999999999994E-4	4.341999999999996E-5	6
3222	1.0	EPI_ISL_417971	6.476799999999993E-4	4.341999999999996E-5	6
3236	1.0	EPI_ISL_418051	6.498599999999993E-4	4.310999999999985E-5	4
3236	1.0	EPI_ISL_427284	6.842499999999993E-4	4.310999999999985E-5	4
3236	1.0	EPI_ISL_427283	6.842499999999993E-4	4.310999999999985E-5	4
3236	1.0	EPI_ISL_418049	6.498599999999993E-4	4.310999999999985E-5	4
3259	1.0	EPI_ISL_420810	8.021599999999991E-4	4.359999999999911E-6	2
3259	1.0	EPI_ISL_420811	8.021599999999991E-4	4.359999999999911E-6	2
3272	1.0	EPI_ISL_426485	6.454999999999994E-4	4.359999999999911E-6	2
3272	1.0	EPI_ISL_426541	6.454999999999994E-4	4.359999999999911E-6	2
3277	1.0	EPI_ISL_426523	6.368099999999996E-4	7.602E-5	3
3277	1.0	EPI_ISL_426500	6.368099999999996E-4	7.602E-5	3
3277	1.0	EPI_ISL_426521	7.084699999999997E-4	7.602E-5	3
3282	1.0	EPI_ISL_427622	6.367699999999996E-4	7.361999999999928E-5	52
3282	1.0	EPI_ISL_416661	6.367699999999996E-4	7.361999999999928E-5	52
3282	1.0	EPI_ISL_418037	6.367699999999996E-4	7.361999999999928E-5	52
3282	1.0	EPI_ISL_427169	6.367699999999996E-4	7.361999999999928E-5	52
3282	1.0	EPI_ISL_424315	8.113699999999991E-4	7.361999999999928E-5	52

bioRxiv preprint doi: https://doi.org/10.1101/2020.11.06.370999; this version posted November 6, 2020. The copyright holder for this preprint (which was not certified by peer review) is the author/funder, who has granted bioRxiv a license to display the preprint in perpetuity. It is made available under aCC-BY 4.0 International license.

Table S4: Clusters identified using a genetic distance threshold within 1% of the distribution of patristic distances within the entire tree. The minimum percentile threshold that maximized the number of clusters was chosen as the optimal threshold by performing multiple clustering runs on randomly sampled patristic distance distributions (1 million for each run) in Phylopart v2 (*46*).

clustername	bootstrap	leafname	branchPath	medianOfDistances	sequencesperCluster
3282	1.0	EPI_ISL_424321	6.367699999999996E-4	7.361999999999928E-5	52
3282	1.0	EPI_ISL_427221	6.558799999999994E-4	7.361999999999928E-5	52
3282	1.0	EPI_ISL_420825	6.520299999999993E-4	7.361999999999928E-5	52
3282	1.0	EPI_ISL_426074	7.397999999999998E-4	7.361999999999928E-5	52
3282	1.0	EPI_ISL_426060	6.367699999999996E-4	7.361999999999928E-5	52
3282	1.0	EPI_ISL_426058	6.798899999999994E-4	7.361999999999928E-5	52
3282	1.0	EPI_ISL_429601	6.367699999999996E-4	7.361999999999928E-5	52
3282	1.0	EPI_ISL_424319	6.563899999999992E-4	7.361999999999928E-5	52
3282	1.0	EPI_ISL_429027	6.367699999999996E-4	7.361999999999928E-5	52
3282	1.0	EPI_ISL_424354	6.781899999999988E-4	7.361999999999928E-5	52
3282	1.0	EPI_ISL_424318	6.367699999999996E-4	7.361999999999928E-5	52
3282	1.0	EPI_ISL_429009	6.367699999999996E-4	7.361999999999928E-5	52
3282	1.0	EPI_ISL_426067	6.411299999999995E-4	7.361999999999928E-5	52
3282	1.0	EPI_ISL_424225	6.842299999999994E-4	7.361999999999928E-5	52
3282	1.0	EPI_ISL_429648	7.131999999999996E-4	7.361999999999928E-5	52
3282	1.0	EPI_ISL_426077	6.367699999999996E-4	7.361999999999928E-5	52
3282	1.0	EPI_ISL_424989	7.046899999999995E-4	7.361999999999928E-5	52
3282	1.0	EPI_ISL_427170	6.367699999999996E-4	7.361999999999928E-5	52
3282	1.0	EPI_ISL_425146	7.38849999999999E-4	7.361999999999928E-5	52
3282	1.0	EPI_ISL_417345	6.433099999999995E-4	7.361999999999928E-5	52
3282	1.0	EPI_ISL_424327	6.367699999999996E-4	7.361999999999928E-5	52
3282	1.0	EPI_ISL_426061	6.798699999999995E-4	7.361999999999928E-5	52
3282	1.0	EPI_ISL_422972	6.454899999999994E-4	7.361999999999928E-5	52
3282	1.0	EPI_ISL_426063	8.54859999999999E-4	7.361999999999928E-5	52
3282	1.0	EPI_ISL_426072	7.016699999999991E-4	7.361999999999928E-5	52
3282	1.0	EPI_ISL_429003	6.367699999999996E-4	7.361999999999928E-5	52

bioRxiv preprint doi: https://doi.org/10.1101/2020.11.06.370999; this version posted November 6, 2020. The copyright holder for this preprint (which was not certified by peer review) is the author/funder, who has granted bioRxiv a license to display the preprint in perpetuity. It is made available under aCC-BY 4.0 International license.

Table S4: Clusters identified using a genetic distance threshold within 1% of the distribution of patristic distances within the entire tree. The minimum percentile threshold that maximized the number of clusters was chosen as the optimal threshold by performing multiple clustering runs on randomly sampled patristic distance distributions (1 million for each run) in Phylopart v2 (*46*).

clustername	bootstrap	leafname	branchPath	medianOfDistances	sequencesperCluster
3282	1.0	EPI_ISL_426073	7.257699999999993E-4	7.361999999999928E-5	52
3282	1.0	EPI_ISL_424176	7.0409999999999999E-4	7.361999999999928E-5	52
3282	1.0	EPI_ISL_418072	6.760099999999988E-4	7.361999999999928E-5	52
3282	1.0	EPI_ISL_426069	6.973099999999992E-4	7.361999999999928E-5	52
3282	1.0	EPI_ISL_424320	6.875499999999994E-4	7.361999999999928E-5	52
3282	1.0	EPI_ISL_426127	6.755099999999996E-4	7.361999999999928E-5	52
3282	1.0	EPI_ISL_424307	6.367699999999996E-4	7.361999999999928E-5	52
3282	1.0	EPI_ISL_430028	6.932799999999992E-4	7.361999999999928E-5	52
3282	1.0	EPI_ISL_426831	7.125799999999988E-4	7.361999999999928E-5	52
3282	1.0	EPI_ISL_426064	7.323499999999991E-4	7.361999999999928E-5	52
3282	1.0	EPI_ISL_428992	7.148699999999995E-4	7.361999999999928E-5	52
3282	1.0	EPI_ISL_426066	6.367699999999996E-4	7.361999999999928E-5	52
3282	1.0	EPI_ISL_429599	7.087599999999995E-4	7.361999999999928E-5	52
3282	1.0	EPI_ISL_426065	6.367699999999996E-4	7.361999999999928E-5	52
3282	1.0	EPI_ISL_429042	7.447799999999989E-4	7.361999999999928E-5	52
3282	1.0	EPI_ISL_426071	6.411299999999996E-4	7.361999999999928E-5	52
3282	1.0	EPI_ISL_417932	6.367699999999996E-4	7.361999999999928E-5	52
3282	1.0	EPI_ISL_429610	6.475699999999994E-4	7.361999999999928E-5	52
3282	1.0	EPI_ISL_418845	6.716499999999989E-4	7.361999999999928E-5	52
3282	1.0	EPI_ISL_424322	6.367699999999996E-4	7.361999999999928E-5	52
3282	1.0	EPI_ISL_429037	7.192299999999994E-4	7.361999999999928E-5	52
3387	1.0	EPI_ISL_426535	7.036899999999998E-4	4.0929999999999786E-5	5
3387	1.0	EPI_ISL_426505	7.036899999999998E-4	4.0929999999999786E-5	5
3387	1.0	EPI_ISL_426520	7.058699999999997E-4	4.0929999999999786E-5	5
3387	1.0	EPI_ISL_426501	6.714799999999997E-4	4.0929999999999786E-5	5
3387	1.0	EPI_ISL_426522	7.058699999999997E-4	4.0929999999999786E-5	5

bioRxiv preprint doi: https://doi.org/10.1101/2020.11.06.370999; this version posted November 6, 2020. The copyright holder for this preprint (which was not certified by peer review) is the author/funder, who has granted bioRxiv a license to display the preprint in perpetuity. It is made available under aCC-BY 4.0 International license.

Table S4: Clusters identified using a genetic distance threshold within 1% of the distribution of patristic distances within the entire tree. The minimum percentile threshold that maximized the number of clusters was chosen as the optimal threshold by performing multiple clustering runs on randomly sampled patristic distance distributions (1 million for each run) in Phylopart v2 (*46*).

clustername	bootstrap	leafname	branchPath	medianOfDistances	sequencesperCluster
3399	1.0	EPI_ISL_429026	6.671399999999998E-4	7.354999999999992E-5	2
3399	1.0	EPI_ISL_428396	6.675699999999997E-4	7.354999999999992E-5	2
3404	1.0	EPI_ISL_427073	6.695199999999996E-4	4.856999999999973E-5	6
3404	1.0	EPI_ISL_426928	6.704499999999996E-4	4.856999999999973E-5	6
3404	1.0	EPI_ISL_426756	6.393199999999995E-4	4.856999999999973E-5	6
3404	1.0	EPI_ISL_426805	7.445399999999996E-4	4.856999999999973E-5	6
3404	1.0	EPI_ISL_419951	6.393199999999995E-4	4.856999999999973E-5	6
3404	1.0	EPI_ISL_426991	6.349599999999996E-4	4.856999999999973E-5	6
3426	1.0	EPI_ISL_420312	6.343199999999996E-4	4.359999999999911E-6	3
3426	1.0	EPI_ISL_429277	6.343199999999996E-4	4.359999999999911E-6	3
3426	1.0	EPI_ISL_429391	6.343199999999996E-4	4.359999999999911E-6	3
3439	1.0	EPI_ISL_429460	5.890399999999999E-4	6.539999999999758E-6	4
3439	1.0	EPI_ISL_429271	5.890399999999999E-4	6.539999999999758E-6	4
3439	1.0	EPI_ISL_418396	5.868599999999999E-4	6.539999999999758E-6	4
3439	1.0	EPI_ISL_427673	5.890399999999999E-4	6.539999999999758E-6	4
3470	1.0	EPI_ISL_421551	5.955899999999997E-4	4.359999999999911E-6	2
3470	1.0	EPI_ISL_422740	5.955899999999997E-4	4.359999999999911E-6	2
3473	1.0	EPI_ISL_430035	6.324799999999997E-4	7.811999999999988E-5	3
3473	1.0	EPI_ISL_417976	6.671699999999997E-4	7.811999999999988E-5	3
3473	1.0	EPI_ISL_419806	5.977699999999996E-4	7.811999999999988E-5	3
3478	1.0	EPI_ISL_421600	5.912299999999998E-4	3.874999999999994E-5	2
3478	1.0	EPI_ISL_419704	6.256199999999998E-4	3.874999999999994E-5	2
3507	1.0	EPI_ISL_426499	7.023699999999998E-4	7.612999999999999E-5	2
3507	1.0	EPI_ISL_426507	6.305999999999997E-4	7.612999999999999E-5	2
3511	0.988	EPI_ISL_417701	5.911799999999997E-4	4.359999999999911E-6	3
3511	0.988	EPI_ISL_427613	5.911799999999997E-4	4.359999999999911E-6	3

bioRxiv preprint doi: https://doi.org/10.1101/2020.11.06.370999; this version posted November 6, 2020. The copyright holder for this preprint (which was not certified by peer review) is the author/funder, who has granted bioRxiv a license to display the preprint in perpetuity. It is made available under aCC-BY 4.0 International license.

Table S4: Clusters identified using a genetic distance threshold within 1% of the distribution of patristic distances within the entire tree. The minimum percentile threshold that maximized the number of clusters was chosen as the optimal threshold by performing multiple clustering runs on randomly sampled patristic distance distributions (1 million for each run) in Phylopart v2 (*46*).

clustername	bootstrap	leafname	branchPath	medianOfDistances	sequencesperCluster
3511	0.988	EPI_ISL_427603	5.911799999999997E-4	4.359999999999911E-6	3
3522	1.0	EPI_ISL_418646	6.364399999999996E-4	8.719999999999822E-6	9
3522	1.0	EPI_ISL_418666	6.342599999999997E-4	8.719999999999822E-6	9
3522	1.0	EPI_ISL_427344	6.298999999999998E-4	8.719999999999822E-6	9
3522	1.0	EPI_ISL_420085	6.364399999999996E-4	8.719999999999822E-6	9
3522	1.0	EPI_ISL_417020	6.364399999999996E-4	8.719999999999822E-6	9
3522	1.0	EPI_ISL_418636	6.320799999999997E-4	8.719999999999822E-6	9
3522	1.0	EPI_ISL_424637	6.364399999999996E-4	8.719999999999822E-6	9
3522	1.0	EPI_ISL_421194	6.708299999999996E-4	8.719999999999822E-6	9
3522	1.0	EPI_ISL_421182	6.364399999999996E-4	8.719999999999822E-6	9
3544	1.0	EPI_ISL_427519	6.240399999999999E-4	4.359999999999911E-6	2
3544	1.0	EPI_ISL_421593	6.240399999999999E-4	4.359999999999911E-6	2
3687	1.0	EPI_ISL_427498	7.531999999999988E-4	4.359999999999911E-6	2
3687	1.0	EPI_ISL_421632	7.531999999999988E-4	4.359999999999911E-6	2
3691	1.0	EPI_ISL_420590	7.83219999999999E-4	3.874E-5	2
3691	1.0	EPI_ISL_420577	7.488399999999989E-4	3.874E-5	2
3726	1.0	EPI_ISL_424943	8.094999999999991E-4	7.534000000000013E-5	6
3726	1.0	EPI_ISL_422503	7.767199999999991E-4	7.534000000000013E-5	6
3726	1.0	EPI_ISL_427585	7.44479999999999E-4	7.534000000000013E-5	6
3726	1.0	EPI_ISL_427631	7.788599999999991E-4	7.534000000000013E-5	6
3726	1.0	EPI_ISL_422510	7.746599999999991E-4	7.534000000000013E-5	6
3726	1.0	EPI_ISL_427588	7.44479999999999E-4	7.534000000000013E-5	6
3739	1.0	EPI_ISL_424935	7.313999999999992E-4	4.359999999999911E-6	2
3739	1.0	EPI_ISL_418190	7.313999999999992E-4	4.359999999999911E-6	2
3742	1.0	EPI_ISL_427629	8.007999999999993E-4	4.0929999999999786E-5	6
3742	1.0	EPI_ISL_427553	7.707699999999992E-4	4.0929999999999786E-5	6

bioRxiv preprint doi: https://doi.org/10.1101/2020.11.06.370999; this version posted November 6, 2020. The copyright holder for this preprint (which was not certified by peer review) is the author/funder, who has granted bioRxiv a license to display the preprint in perpetuity. It is made available under aCC-BY 4.0 International license.

Table S4: Clusters identified using a genetic distance threshold within 1% of the distribution of patristic distances within the entire tree. The minimum percentile threshold that maximized the number of clusters was chosen as the optimal threshold by performing multiple clustering runs on randomly sampled patristic distance distributions (1 million for each run) in Phylopart v2 (*46*).

clustername	bootstrap	leafname	branchPath	medianOfDistances	sequencesperCluster
3742	1.0	EPI_ISL_421422	7.707699999999992E-4	4.0929999999999786E-5	6
3742	1.0	EPI_ISL_421388	7.707699999999992E-4	4.0929999999999786E-5	6
3742	1.0	EPI_ISL_427514	8.029799999999993E-4	4.0929999999999786E-5	6
3742	1.0	EPI_ISL_421389	7.707699999999992E-4	4.0929999999999786E-5	6
3791	1.0	EPI_ISL_420574	7.578899999999995E-4	3.956999999999984E-5	2
3791	1.0	EPI_ISL_421581	7.226799999999994E-4	3.956999999999984E-5	2
3801	1.0	EPI_ISL_418973	7.636099999999993E-4	4.310999999999985E-5	10
3801	1.0	EPI_ISL_421583	7.313999999999992E-4	4.310999999999985E-5	10
3801	1.0	EPI_ISL_420581	7.313999999999992E-4	4.310999999999985E-5	10
3801	1.0	EPI_ISL_426618	7.614299999999994E-4	4.310999999999985E-5	10
3801	1.0	EPI_ISL_420297	7.313999999999992E-4	4.310999999999985E-5	10
3801	1.0	EPI_ISL_428800	7.964099999999996E-4	4.310999999999985E-5	10
3801	1.0	EPI_ISL_422512	7.248599999999994E-4	4.310999999999985E-5	10
3801	1.0	EPI_ISL_421421	7.313999999999992E-4	4.310999999999985E-5	10
3801	1.0	EPI_ISL_424953	7.570699999999994E-4	4.310999999999985E-5	10
3801	1.0	EPI_ISL_421597	7.313999999999992E-4	4.310999999999985E-5	10
3894	1.0	EPI_ISL_427064	7.843599999999997E-4	3.910000000000003E-5	2
3894	1.0	EPI_ISL_427065	7.496199999999997E-4	3.910000000000003E-5	2
3915	1.0	EPI_ISL_422906	7.519599999999995E-4	3.962999999999992E-5	2
3915	1.0	EPI_ISL_422703	7.166899999999995E-4	3.962999999999992E-5	2
3930	0.944	EPI_ISL_415485	7.559599999999995E-4	3.874E-5	3
3930	0.944	EPI_ISL_415474	7.215799999999994E-4	3.874E-5	3
3930	0.944	EPI_ISL_415473	7.215799999999994E-4	3.874E-5	3
3936	1.0	EPI_ISL_427501	6.307299999999997E-4	7.390000000000001E-5	2
3936	1.0	EPI_ISL_424941	6.299499999999998E-4	7.390000000000001E-5	2
3939	1.0	EPI_ISL_426939	7.040999999999997E-4	3.987999999999995E-5	2

bioRxiv preprint doi: https://doi.org/10.1101/2020.11.06.370999; this version posted November 6, 2020. The copyright holder for this preprint (which was not certified by peer review) is the author/funder, who has granted bioRxiv a license to display the preprint in perpetuity. It is made available under aCC-BY 4.0 International license.

Table S4: Clusters identified using a genetic distance threshold within 1% of the distribution of patristic distances within the entire tree. The minimum percentile threshold that maximized the number of clusters was chosen as the optimal threshold by performing multiple clustering runs on randomly sampled patristic distance distributions (1 million for each run) in Phylopart v2 (*46*).

clustername	bootstrap	leafname	branchPath	medianOfDistances	sequencesperCluster
3939	1.0	EPI_ISL_426657	6.685799999999997E-4	3.987999999999995E-5	2
3956	1.0	EPI_ISL_421346	5.977499999999996E-4	4.359999999999911E-6	6
3956	1.0	EPI_ISL_421545	5.977499999999996E-4	4.359999999999911E-6	6
3956	1.0	EPI_ISL_421544	5.977499999999996E-4	4.359999999999911E-6	6
3956	1.0	EPI_ISL_421347	6.308899999999997E-4	4.359999999999911E-6	6
3956	1.0	EPI_ISL_421543	5.977499999999996E-4	4.359999999999911E-6	6
3956	1.0	EPI_ISL_421553	5.977499999999996E-4	4.359999999999911E-6	6
3967	1.0	EPI_ISL_429517	6.605999999999999E-4	7.312000000000009E-5	2
3967	1.0	EPI_ISL_429585	6.605999999999999E-4	7.312000000000009E-5	2
3988	1.0	EPI_ISL_420761	6.608199999999999E-4	3.895999999999999E-5	2
3988	1.0	EPI_ISL_422408	6.262199999999998E-4	3.895999999999999E-5	2
3995	1.0	EPI_ISL_429067	5.912199999999998E-4	4.359999999999911E-6	2
3995	1.0	EPI_ISL_429989	5.912199999999998E-4	4.359999999999911E-6	2
4003	1.0	EPI_ISL_420079	0.00103186	5.297999999999978E-5	2
4003	1.0	EPI_ISL_420071	9.8324E-4	5.297999999999978E-5	2
4056	1.0	EPI_ISL_429403	6.366399999999996E-4	6.539999999999758E-6	3
4056	1.0	EPI_ISL_429267	6.388199999999996E-4	6.539999999999758E-6	3
4056	1.0	EPI_ISL_429265	6.388199999999996E-4	6.539999999999758E-6	3
4061	1.0	EPI_ISL_429361	5.999399999999997E-4	4.359999999999911E-6	2
4061	1.0	EPI_ISL_429416	5.999399999999997E-4	4.359999999999911E-6	2
4064	1.0	EPI_ISL_427639	6.321399999999998E-4	3.874E-5	2
4064	1.0	EPI_ISL_429441	5.977599999999997E-4	3.874E-5	2
4108	1.0	EPI_ISL_424335	5.955799999999997E-4	4.0919999999999845E-5	3
4108	1.0	EPI_ISL_429794	6.321399999999998E-4	4.0919999999999845E-5	3
4108	1.0	EPI_ISL_429722	6.321399999999998E-4	4.0919999999999845E-5	3
4115	1.0	EPI_ISL_427250	6.299599999999997E-4	4.0919999999999845E-5	6

bioRxiv preprint doi: https://doi.org/10.1101/2020.11.06.370999; this version posted November 6, 2020. The copyright holder for this preprint (which was not certified by peer review) is the author/funder, who has granted bioRxiv a license to display the preprint in perpetuity. It is made available under aCC-BY 4.0 International license.

Table S4: Clusters identified using a genetic distance threshold within 1% of the distribution of patristic distances within the entire tree. The minimum percentile threshold that maximized the number of clusters was chosen as the optimal threshold by performing multiple clustering runs on randomly sampled patristic distance distributions (1 million for each run) in Phylopart v2 (*46*).

clustername	bootstrap	leafname	branchPath	medianOfDistances	sequencesperCluster
4115	1.0	EPI_ISL_429290	6.455199999999998E-4	4.0919999999999845E-5	6
4115	1.0	EPI_ISL_426687	5.977599999999997E-4	4.0919999999999845E-5	6
4115	1.0	EPI_ISL_429275	5.977599999999997E-4	4.0919999999999845E-5	6
4115	1.0	EPI_ISL_429382	5.977599999999997E-4	4.0919999999999845E-5	6
4115	1.0	EPI_ISL_429274	5.977599999999997E-4	4.0919999999999845E-5	6
4129	1.0	EPI_ISL_422508	6.693399999999997E-4	3.875999999999988E-5	2
4129	1.0	EPI_ISL_421590	6.349399999999996E-4	3.875999999999988E-5	2
4136	1.0	EPI_ISL_424491	6.349399999999996E-4	4.359999999999911E-6	2
4136	1.0	EPI_ISL_424409	6.349399999999996E-4	4.359999999999911E-6	2
4142	1.0	EPI_ISL_424314	6.517599999999994E-4	8.430999999999868E-5	185
4142	1.0	EPI_ISL_427196	6.282799999999991E-4	8.430999999999868E-5	185
4142	1.0	EPI_ISL_418879	7.438999999999997E-4	8.430999999999868E-5	185
4142	1.0	EPI_ISL_426093	6.604799999999992E-4	8.430999999999868E-5	185
4142	1.0	EPI_ISL_426079	6.324799999999991E-4	8.430999999999868E-5	185
4142	1.0	EPI_ISL_418876	6.439299999999988E-4	8.430999999999868E-5	185
4142	1.0	EPI_ISL_424193	6.413599999999988E-4	8.430999999999868E-5	185
4142	1.0	EPI_ISL_427256	6.517599999999994E-4	8.430999999999868E-5	185
4142	1.0	EPI_ISL_424277	8.507399999999987E-4	8.430999999999868E-5	185
4142	1.0	EPI_ISL_422967	6.413599999999988E-4	8.430999999999868E-5	185
4142	1.0	EPI_ISL_422998	6.217399999999992E-4	8.430999999999868E-5	185
4142	1.0	EPI_ISL_427181	6.861399999999995E-4	8.430999999999868E-5	185
4142	1.0	EPI_ISL_418904	6.604799999999992E-4	8.430999999999868E-5	185
4142	1.0	EPI_ISL_426080	6.348199999999989E-4	8.430999999999868E-5	185
4142	1.0	EPI_ISL_429615	6.368399999999996E-4	8.430999999999868E-5	185
4142	1.0	EPI_ISL_418900	6.435399999999988E-4	8.430999999999868E-5	185
4142	1.0	EPI_ISL_424331	6.473999999999995E-4	8.430999999999868E-5	185

bioRxiv preprint doi: https://doi.org/10.1101/2020.11.06.370999; this version posted November 6, 2020. The copyright holder for this preprint (which was not certified by peer review) is the author/funder, who has granted bioRxiv a license to display the preprint in perpetuity. It is made available under aCC-BY 4.0 International license.

Table S4: Clusters identified using a genetic distance threshold within 1% of the distribution of patristic distances within the entire tree. The minimum percentile threshold that maximized the number of clusters was chosen as the optimal threshold by performing multiple clustering runs on randomly sampled patristic distance distributions (1 million for each run) in Phylopart v2 (*46*).

clustername	bootstrap	leafname	branchPath	medianOfDistances	sequencesperCluster
4142	1.0	EPI_ISL_423024	6.282799999999991E-4	8.430999999999868E-5	185
4142	1.0	EPI_ISL_429653	6.30459999999999E-4	8.430999999999868E-5	185
4142	1.0	EPI_ISL_429359	7.207199999999996E-4	8.430999999999868E-5	185
4142	1.0	EPI_ISL_427254	6.517599999999994E-4	8.430999999999868E-5	185
4142	1.0	EPI_ISL_424242	7.342199999999992E-4	8.430999999999868E-5	185
4142	1.0	EPI_ISL_427200	6.582999999999993E-4	8.430999999999868E-5	185
4142	1.0	EPI_ISL_426091	6.413599999999988E-4	8.430999999999868E-5	185
4142	1.0	EPI_ISL_422979	6.427499999999988E-4	8.430999999999868E-5	185
4142	1.0	EPI_ISL_424205	6.609799999999984E-4	8.430999999999868E-5	185
4142	1.0	EPI_ISL_427202	6.539399999999993E-4	8.430999999999868E-5	185
4142	1.0	EPI_ISL_426087	6.738299999999989E-4	8.430999999999868E-5	185
4142	1.0	EPI_ISL_417454	6.71379999999999E-4	8.430999999999868E-5	185
4142	1.0	EPI_ISL_429631	6.413599999999988E-4	8.430999999999868E-5	185
4142	1.0	EPI_ISL_417451	6.32639999999999E-4	8.430999999999868E-5	185
4142	1.0	EPI_ISL_427209	6.839699999999995E-4	8.430999999999868E-5	185
4142	1.0	EPI_ISL_424200	6.32639999999999E-4	8.430999999999868E-5	185
4142	1.0	EPI_ISL_416647	6.369999999999989E-4	8.430999999999868E-5	185
4142	1.0	EPI_ISL_429608	6.299599999999998E-4	8.430999999999868E-5	185
4142	1.0	EPI_ISL_427215	6.604799999999992E-4	8.430999999999868E-5	185
4142	1.0	EPI_ISL_418929	6.32639999999999E-4	8.430999999999868E-5	185
4142	1.0	EPI_ISL_427230	6.861799999999995E-4	8.430999999999868E-5	185
4142	1.0	EPI_ISL_418924	6.413599999999988E-4	8.430999999999868E-5	185
4142	1.0	EPI_ISL_427255	6.517599999999994E-4	8.430999999999868E-5	185
4142	1.0	EPI_ISL_426081	6.71379999999999E-4	8.430999999999868E-5	185
4142	1.0	EPI_ISL_422978	6.239199999999992E-4	8.430999999999868E-5	185
4142	1.0	EPI_ISL_418923	6.369999999999989E-4	8.430999999999868E-5	185

bioRxiv preprint doi: https://doi.org/10.1101/2020.11.06.370999; this version posted November 6, 2020. The copyright holder for this preprint (which was not certified by peer review) is the author/funder, who has granted bioRxiv a license to display the preprint in perpetuity. It is made available under aCC-BY 4.0 International license.

Table S4: Clusters identified using a genetic distance threshold within 1% of the distribution of patristic distances within the entire tree. The minimum percentile threshold that maximized the number of clusters was chosen as the optimal threshold by performing multiple clustering runs on randomly sampled patristic distance distributions (1 million for each run) in Phylopart v2 (*46*).

clustername	bootstrap	leafname	branchPath	medianOfDistances	sequencesperCluster
4142	1.0	EPI_ISL_424333	6.343299999999997E-4	8.430999999999868E-5	185
4142	1.0	EPI_ISL_416708	6.282799999999991E-4	8.430999999999868E-5	185
4142	1.0	EPI_ISL_418932	6.670199999999991E-4	8.430999999999868E-5	185
4142	1.0	EPI_ISL_424202	6.413599999999988E-4	8.430999999999868E-5	185
4142	1.0	EPI_ISL_416691	6.626599999999992E-4	8.430999999999868E-5	185
4142	1.0	EPI_ISL_416719	6.413599999999988E-4	8.430999999999868E-5	185
4142	1.0	EPI_ISL_424204	6.604799999999992E-4	8.430999999999868E-5	185
4142	1.0	EPI_ISL_424309	6.71379999999999E-4	8.430999999999868E-5	185
4142	1.0	EPI_ISL_429607	9.494899999999994E-4	8.430999999999868E-5	185
4142	1.0	EPI_ISL_426104	6.260999999999991E-4	8.430999999999868E-5	185
4142	1.0	EPI_ISL_424310	6.173799999999993E-4	8.430999999999868E-5	185
4142	1.0	EPI_ISL_424227	6.495799999999994E-4	8.430999999999868E-5	185
4142	1.0	EPI_ISL_427189	6.604799999999992E-4	8.430999999999868E-5	185
4142	1.0	EPI_ISL_427191	6.413599999999988E-4	8.430999999999868E-5	185
4142	1.0	EPI_ISL_427210	7.363999999999991E-4	8.430999999999868E-5	185
4142	1.0	EPI_ISL_422983	7.335299999999992E-4	8.430999999999868E-5	185
4142	1.0	EPI_ISL_424221	6.500799999999986E-4	8.430999999999868E-5	185
4142	1.0	EPI_ISL_429600	6.67869999999999E-4	8.430999999999868E-5	185
4142	1.0	EPI_ISL_418913	6.042999999999996E-4	8.430999999999868E-5	185
4142	1.0	EPI_ISL_424203	6.604799999999992E-4	8.430999999999868E-5	185
4142	1.0	EPI_ISL_417342	6.413599999999988E-4	8.430999999999868E-5	185
4142	1.0	EPI_ISL_424178	6.745299999999989E-4	8.430999999999868E-5	185
4142	1.0	EPI_ISL_424302	6.413599999999988E-4	8.430999999999868E-5	185
4142	1.0	EPI_ISL_427239	7.599099999999995E-4	8.430999999999868E-5	185
4142	1.0	EPI_ISL_424238	6.72479999999999E-4	8.430999999999868E-5	185
4142	1.0	EPI_ISL_422985	6.32639999999999E-4	8.430999999999868E-5	185

bioRxiv preprint doi: https://doi.org/10.1101/2020.11.06.370999; this version posted November 6, 2020. The copyright holder for this preprint (which was not certified by peer review) is the author/funder, who has granted bioRxiv a license to display the preprint in perpetuity. It is made available under aCC-BY 4.0 International license.

Table S4: Clusters identified using a genetic distance threshold within 1% of the distribution of patristic distances within the entire tree. The minimum percentile threshold that maximized the number of clusters was chosen as the optimal threshold by performing multiple clustering runs on randomly sampled patristic distance distributions (1 million for each run) in Phylopart v2 (*46*).

clustername	bootstrap	leafname	branchPath	medianOfDistances	sequencesperCluster
4142	1.0	EPI_ISL_427236	6.883999999999995E-4	8.430999999999868E-5	185
4142	1.0	EPI_ISL_416707	6.348199999999989E-4	8.430999999999868E-5	185
4142	1.0	EPI_ISL_424305	6.631599999999984E-4	8.430999999999868E-5	185
4142	1.0	EPI_ISL_418878	7.788199999999999E-4	8.430999999999868E-5	185
4142	1.0	EPI_ISL_424208	6.364999999999997E-4	8.430999999999868E-5	185
4142	1.0	EPI_ISL_424312	6.413599999999988E-4	8.430999999999868E-5	185
4142	1.0	EPI_ISL_427225	7.579399999999995E-4	8.430999999999868E-5	185
4142	1.0	EPI_ISL_416693	6.648399999999991E-4	8.430999999999868E-5	185
4142	1.0	EPI_ISL_416653	6.631599999999984E-4	8.430999999999868E-5	185
4142	1.0	EPI_ISL_426116	6.326399999999999E-4	8.430999999999868E-5	185
4142	1.0	EPI_ISL_423001	6.691999999999999E-4	8.430999999999868E-5	185
4142	1.0	EPI_ISL_426115	7.705699999999992E-4	8.430999999999868E-5	185
4142	1.0	EPI_ISL_429622	6.648399999999991E-4	8.430999999999868E-5	185
4142	1.0	EPI_ISL_424245	6.348199999999989E-4	8.430999999999868E-5	185
4142	1.0	EPI_ISL_429617	6.713799999999999E-4	8.430999999999868E-5	185
4142	1.0	EPI_ISL_427194	6.495799999999994E-4	8.430999999999868E-5	185
4142	1.0	EPI_ISL_424324	6.696599999999991E-4	8.430999999999868E-5	185
4142	1.0	EPI_ISL_427247	6.604799999999992E-4	8.430999999999868E-5	185
4142	1.0	EPI_ISL_424259	6.413599999999988E-4	8.430999999999868E-5	185
4142	1.0	EPI_ISL_426134	6.430499999999995E-4	8.430999999999868E-5	185
4142	1.0	EPI_ISL_424246	6.435399999999988E-4	8.430999999999868E-5	185
4142	1.0	EPI_ISL_429654	6.517599999999994E-4	8.430999999999868E-5	185
4142	1.0	EPI_ISL_423018	6.413599999999988E-4	8.430999999999868E-5	185
4142	1.0	EPI_ISL_424229	6.635499999999991E-4	8.430999999999868E-5	185
4142	1.0	EPI_ISL_424212	6.386799999999997E-4	8.430999999999868E-5	185
4142	1.0	EPI_ISL_429651	6.413599999999988E-4	8.430999999999868E-5	185

bioRxiv preprint doi: https://doi.org/10.1101/2020.11.06.370999; this version posted November 6, 2020. The copyright holder for this preprint (which was not certified by peer review) is the author/funder, who has granted bioRxiv a license to display the preprint in perpetuity. It is made available under aCC-BY 4.0 International license.

Table S4: Clusters identified using a genetic distance threshold within 1% of the distribution of patristic distances within the entire tree. The minimum percentile threshold that maximized the number of clusters was chosen as the optimal threshold by performing multiple clustering runs on randomly sampled patristic distance distributions (1 million for each run) in Phylopart v2 (*46*).

clustername	bootstrap	leafname	branchPath	medianOfDistances	sequencesperCluster
4142	1.0	EPI_ISL_422970	6.30459999999999E-4	8.430999999999868E-5	185
4142	1.0	EPI_ISL_429652	6.380799999999991E-4	8.430999999999868E-5	185
4142	1.0	EPI_ISL_424256	6.824099999999995E-4	8.430999999999868E-5	185
4142	1.0	EPI_ISL_424340	6.413599999999988E-4	8.430999999999868E-5	185
4142	1.0	EPI_ISL_424214	6.926399999999993E-4	8.430999999999868E-5	185
4142	1.0	EPI_ISL_422976	6.413599999999988E-4	8.430999999999868E-5	185
4142	1.0	EPI_ISL_429614	6.71399999999999E-4	8.430999999999868E-5	185
4142	1.0	EPI_ISL_416438	6.413599999999988E-4	8.430999999999868E-5	185
4142	1.0	EPI_ISL_427260	6.413599999999988E-4	8.430999999999868E-5	185
4142	1.0	EPI_ISL_427207	6.457199999999987E-4	8.430999999999868E-5	185
4142	1.0	EPI_ISL_418054	6.604799999999992E-4	8.430999999999868E-5	185
4142	1.0	EPI_ISL_424235	7.024199999999991E-4	8.430999999999868E-5	185
4142	1.0	EPI_ISL_427180	6.30459999999999E-4	8.430999999999868E-5	185
4142	1.0	EPI_ISL_429637	6.582999999999993E-4	8.430999999999868E-5	185
4142	1.0	EPI_ISL_417382	6.32639999999999E-4	8.430999999999868E-5	185
4142	1.0	EPI_ISL_427216	6.413599999999988E-4	8.430999999999868E-5	185
4142	1.0	EPI_ISL_418951	6.413599999999988E-4	8.430999999999868E-5	185
4142	1.0	EPI_ISL_427222	6.474099999999994E-4	8.430999999999868E-5	185
4142	1.0	EPI_ISL_424270	6.817899999999995E-4	8.430999999999868E-5	185
4142	1.0	EPI_ISL_427199	6.413599999999988E-4	8.430999999999868E-5	185
4142	1.0	EPI_ISL_423010	6.413599999999988E-4	8.430999999999868E-5	185
4142	1.0	EPI_ISL_416698	6.260999999999991E-4	8.430999999999868E-5	185
4142	1.0	EPI_ISL_429655	6.779299999999988E-4	8.430999999999868E-5	185
4142	1.0	EPI_ISL_417455	6.217399999999992E-4	8.430999999999868E-5	185
4142	1.0	EPI_ISL_427228	6.282799999999991E-4	8.430999999999868E-5	185
4142	1.0	EPI_ISL_427234	9.075999999999995E-4	8.430999999999868E-5	185

bioRxiv preprint doi: https://doi.org/10.1101/2020.11.06.370999; this version posted November 6, 2020. The copyright holder for this preprint (which was not certified by peer review) is the author/funder, who has granted bioRxiv a license to display the preprint in perpetuity. It is made available under aCC-BY 4.0 International license.

Table S4: Clusters identified using a genetic distance threshold within 1% of the distribution of patristic distances within the entire tree. The minimum percentile threshold that maximized the number of clusters was chosen as the optimal threshold by performing multiple clustering runs on randomly sampled patristic distance distributions (1 million for each run) in Phylopart v2 (*46*).

clustername	bootstrap	leafname	branchPath	medianOfDistances	sequencesperCluster
4142	1.0	EPI_ISL_418032	6.544399999999985E-4	8.430999999999868E-5	185
4142	1.0	EPI_ISL_422981	6.413599999999988E-4	8.430999999999868E-5	185
4142	1.0	EPI_ISL_424337	6.71379999999999E-4	8.430999999999868E-5	185
4142	1.0	EPI_ISL_427197	6.413599999999988E-4	8.430999999999868E-5	185
4142	1.0	EPI_ISL_424189	6.566199999999985E-4	8.430999999999868E-5	185
4142	1.0	EPI_ISL_424197	7.042599999999991E-4	8.430999999999868E-5	185
4142	1.0	EPI_ISL_422969	6.239199999999992E-4	8.430999999999868E-5	185
4142	1.0	EPI_ISL_416704	6.611999999999992E-4	8.430999999999868E-5	185
4142	1.0	EPI_ISL_426125	7.766899999999989E-4	8.430999999999868E-5	185
4142	1.0	EPI_ISL_418068	8.666199999999987E-4	8.430999999999868E-5	185
4142	1.0	EPI_ISL_424184	6.413599999999988E-4	8.430999999999868E-5	185
4142	1.0	EPI_ISL_418067	6.30459999999999E-4	8.430999999999868E-5	185
4142	1.0	EPI_ISL_416434	6.670199999999991E-4	8.430999999999868E-5	185
4142	1.0	EPI_ISL_418053	6.413599999999988E-4	8.430999999999868E-5	185
4142	1.0	EPI_ISL_429602	6.604899999999991E-4	8.430999999999868E-5	185
4142	1.0	EPI_ISL_416452	6.343599999999997E-4	8.430999999999868E-5	185
4142	1.0	EPI_ISL_423031	6.304699999999991E-4	8.430999999999868E-5	185
4142	1.0	EPI_ISL_418933	6.413599999999988E-4	8.430999999999868E-5	185
4142	1.0	EPI_ISL_424251	6.413599999999988E-4	8.430999999999868E-5	185
4142	1.0	EPI_ISL_426103	6.369999999999989E-4	8.430999999999868E-5	185
4142	1.0	EPI_ISL_426112	6.413599999999988E-4	8.430999999999868E-5	185
4142	1.0	EPI_ISL_418081	6.604799999999992E-4	8.430999999999868E-5	185
4142	1.0	EPI_ISL_418880	6.478999999999987E-4	8.430999999999868E-5	185
4142	1.0	EPI_ISL_424190	6.369999999999989E-4	8.430999999999868E-5	185
4142	1.0	EPI_ISL_416716	6.413599999999988E-4	8.430999999999868E-5	185
4142	1.0	EPI_ISL_426515	6.670199999999991E-4	8.430999999999868E-5	185

bioRxiv preprint doi: https://doi.org/10.1101/2020.11.06.370999; this version posted November 6, 2020. The copyright holder for this preprint (which was not certified by peer review) is the author/funder, who has granted bioRxiv a license to display the preprint in perpetuity. It is made available under aCC-BY 4.0 International license.

Table S4: Clusters identified using a genetic distance threshold within 1% of the distribution of patristic distances within the entire tree. The minimum percentile threshold that maximized the number of clusters was chosen as the optimal threshold by performing multiple clustering runs on randomly sampled patristic distance distributions (1 million for each run) in Phylopart v2 (*46*).

clustername	bootstrap	leafname	branchPath	medianOfDistances	sequencesperCluster
4142	1.0	EPI_ISL_417341	6.260999999999991E-4	8.430999999999868E-5	185
4142	1.0	EPI_ISL_415625	6.587999999999984E-4	8.430999999999868E-5	185
4142	1.0	EPI_ISL_427246	6.670199999999991E-4	8.430999999999868E-5	185
4142	1.0	EPI_ISL_424211	6.30459999999999E-4	8.430999999999868E-5	185
4142	1.0	EPI_ISL_429635	6.495799999999994E-4	8.430999999999868E-5	185
4142	1.0	EPI_ISL_426113	6.583099999999992E-4	8.430999999999868E-5	185
4142	1.0	EPI_ISL_418875	6.32639999999999E-4	8.430999999999868E-5	185
4142	1.0	EPI_ISL_424316	6.517599999999994E-4	8.430999999999868E-5	185
4142	1.0	EPI_ISL_416699	6.130199999999994E-4	8.430999999999868E-5	185
4142	1.0	EPI_ISL_414616	6.391799999999989E-4	8.430999999999868E-5	185
4142	1.0	EPI_ISL_426135	6.413599999999988E-4	8.430999999999868E-5	185
4142	1.0	EPI_ISL_418894	6.108399999999994E-4	8.430999999999868E-5	185
4142	1.0	EPI_ISL_427205	6.413599999999988E-4	8.430999999999868E-5	185
4142	1.0	EPI_ISL_427253	6.861499999999995E-4	8.430999999999868E-5	185
4142	1.0	EPI_ISL_422977	6.517599999999994E-4	8.430999999999868E-5	185
4142	1.0	EPI_ISL_429613	6.32639999999999E-4	8.430999999999868E-5	185
4142	1.0	EPI_ISL_427241	6.408599999999996E-4	8.430999999999868E-5	185
4142	1.0	EPI_ISL_424183	6.413599999999988E-4	8.430999999999868E-5	185
4142	1.0	EPI_ISL_429597	6.384699999999994E-4	8.430999999999868E-5	185
4142	1.0	EPI_ISL_424313	6.413599999999988E-4	8.430999999999868E-5	185
4142	1.0	EPI_ISL_415597	6.413599999999988E-4	8.430999999999868E-5	185
4142	1.0	EPI_ISL_427188	6.604799999999992E-4	8.430999999999868E-5	185
4142	1.0	EPI_ISL_423032	6.992199999999992E-4	8.430999999999868E-5	185
4142	1.0	EPI_ISL_427224	8.001599999999994E-4	8.430999999999868E-5	185
4142	1.0	EPI_ISL_416724	6.260999999999991E-4	8.430999999999868E-5	185
4142	1.0	EPI_ISL_427242	6.861699999999994E-4	8.430999999999868E-5	185

bioRxiv preprint doi: https://doi.org/10.1101/2020.11.06.370999; this version posted November 6, 2020. The copyright holder for this preprint (which was not certified by peer review) is the author/funder, who has granted bioRxiv a license to display the preprint in perpetuity. It is made available under aCC-BY 4.0 International license.

Table S4: Clusters identified using a genetic distance threshold within 1% of the distribution of patristic distances within the entire tree. The minimum percentile threshold that maximized the number of clusters was chosen as the optimal threshold by performing multiple clustering runs on randomly sampled patristic distance distributions (1 million for each run) in Phylopart v2 (*46*).

clustername	bootstrap	leafname	branchPath	medianOfDistances	sequencesperCluster
4142	1.0	EPI_ISL_429609	6.413599999999988E-4	8.430999999999868E-5	185
4142	1.0	EPI_ISL_424334	6.539399999999993E-4	8.430999999999868E-5	185
4142	1.0	EPI_ISL_424274	7.530899999999996E-4	8.430999999999868E-5	185
4142	1.0	EPI_ISL_422996	6.151999999999993E-4	8.430999999999868E-5	185
4142	1.0	EPI_ISL_418073	6.413599999999988E-4	8.430999999999868E-5	185
4142	1.0	EPI_ISL_426109	6.477299999999995E-4	8.430999999999868E-5	185
4142	1.0	EPI_ISL_426085	6.457199999999987E-4	8.430999999999868E-5	185
4142	1.0	EPI_ISL_416729	6.282799999999991E-4	8.430999999999868E-5	185
4142	1.0	EPI_ISL_418953	6.71379999999999E-4	8.430999999999868E-5	185
4142	1.0	EPI_ISL_416648	6.413599999999988E-4	8.430999999999868E-5	185
4142	1.0	EPI_ISL_416725	6.435399999999988E-4	8.430999999999868E-5	185
4142	1.0	EPI_ISL_418033	6.522599999999986E-4	8.430999999999868E-5	185
4511	1.0	EPI_ISL_421420	5.737800000000002E-4	7.600999999999984E-5	3
4511	1.0	EPI_ISL_429642	6.103400000000002E-4	7.600999999999984E-5	3
4511	1.0	EPI_ISL_427165	6.819900000000003E-4	7.600999999999984E-5	3
4518	1.0	EPI_ISL_423030	5.759500000000001E-4	4.359999999999911E-6	2
4518	1.0	EPI_ISL_427217	5.759500000000001E-4	4.359999999999911E-6	2
4522	1.0	EPI_ISL_424186	7.203600000000001E-4	8.020999999999983E-5	7
4522	1.0	EPI_ISL_429603	6.5184E-4	8.020999999999983E-5	7
4522	1.0	EPI_ISL_427267	6.840300000000001E-4	8.020999999999983E-5	7
4522	1.0	EPI_ISL_427192	6.5184E-4	8.020999999999983E-5	7
4522	1.0	EPI_ISL_424341	6.846E-4	8.020999999999983E-5	7
4522	1.0	EPI_ISL_418954	6.1311E-4	8.020999999999983E-5	7
4522	1.0	EPI_ISL_427620	6.474800000000001E-4	8.020999999999983E-5	7
4566	1.0	EPI_ISL_420608	5.307000000000002E-4	4.359999999999911E-6	3
4566	1.0	EPI_ISL_418430	5.307000000000002E-4	4.359999999999911E-6	3

bioRxiv preprint doi: https://doi.org/10.1101/2020.11.06.370999; this version posted November 6, 2020. The copyright holder for this preprint (which was not certified by peer review) is the author/funder, who has granted bioRxiv a license to display the preprint in perpetuity. It is made available under aCC-BY 4.0 International license.

Table S4: Clusters identified using a genetic distance threshold within 1% of the distribution of patristic distances within the entire tree. The minimum percentile threshold that maximized the number of clusters was chosen as the optimal threshold by performing multiple clustering runs on randomly sampled patristic distance distributions (1 million for each run) in Phylopart v2 (*46*).

clustername	bootstrap	leafname	branchPath	medianOfDistances	sequencesperCluster
4566	1.0	EPI_ISL_419173	5.307000000000002E-4	4.359999999999911E-6	3
4594	1.0	EPI_ISL_418413	5.503199999999999E-4	3.872999999999984E-5	2
4594	1.0	EPI_ISL_420615	5.846899999999999E-4	3.872999999999984E-5	2
4612	1.0	EPI_ISL_420617	5.372400000000001E-4	3.881999999999996E-5	2
4612	1.0	EPI_ISL_416758	5.717000000000002E-4	3.881999999999996E-5	2
4625	1.0	EPI_ISL_429669	5.759600000000001E-4	3.87199999999999E-5	2
4625	1.0	EPI_ISL_424929	5.416E-4	3.87199999999999E-5	2
4628	1.0	EPI_ISL_419172	5.4194E-4	4.359999999999911E-6	2
4628	1.0	EPI_ISL_419171	5.4194E-4	4.359999999999911E-6	2
4640	1.0	EPI_ISL_424023	6.1951E-4	8.617999999999972E-5	63
4640	1.0	EPI_ISL_424150	6.325899999999997E-4	8.617999999999972E-5	63
4640	1.0	EPI_ISL_423400	6.952999999999992E-4	8.617999999999972E-5	63
4640	1.0	EPI_ISL_423261	6.822199999999994E-4	8.617999999999972E-5	63
4640	1.0	EPI_ISL_418701	6.522099999999993E-4	8.617999999999972E-5	63
4640	1.0	EPI_ISL_421791	6.969699999999999E-4	8.617999999999972E-5	63
4640	1.0	EPI_ISL_423498	7.057099999999997E-4	8.617999999999972E-5	63
4640	1.0	EPI_ISL_424022	6.260499999999998E-4	8.617999999999972E-5	63
4640	1.0	EPI_ISL_424153	6.413099999999995E-4	8.617999999999972E-5	63
4640	1.0	EPI_ISL_423262	6.887599999999993E-4	8.617999999999972E-5	63
4640	1.0	EPI_ISL_423965	6.282299999999998E-4	8.617999999999972E-5	63
4640	1.0	EPI_ISL_421778	6.756799999999996E-4	8.617999999999972E-5	63
4640	1.0	EPI_ISL_423105	6.522099999999993E-4	8.617999999999972E-5	63
4640	1.0	EPI_ISL_423966	6.391299999999996E-4	8.617999999999972E-5	63
4640	1.0	EPI_ISL_423988	6.238699999999999E-4	8.617999999999972E-5	63
4640	1.0	EPI_ISL_423733	7.01839999999999E-4	8.617999999999972E-5	63
4640	1.0	EPI_ISL_419811	7.193799999999995E-4	8.617999999999972E-5	63

bioRxiv preprint doi: https://doi.org/10.1101/2020.11.06.370999; this version posted November 6, 2020. The copyright holder for this preprint (which was not certified by peer review) is the author/funder, who has granted bioRxiv a license to display the preprint in perpetuity. It is made available under aCC-BY 4.0 International license.

Table S4: Clusters identified using a genetic distance threshold within 1% of the distribution of patristic distances within the entire tree. The minimum percentile threshold that maximized the number of clusters was chosen as the optimal threshold by performing multiple clustering runs on randomly sampled patristic distance distributions (1 million for each run) in Phylopart v2 (*46*).

clustername	bootstrap	leafname	branchPath	medianOfDistances	sequencesperCluster
4640	1.0	EPI_ISL_420724	6.691399999999997E-4	8.617999999999972E-5	63
4640	1.0	EPI_ISL_423732	6.778599999999995E-4	8.617999999999972E-5	63
4640	1.0	EPI_ISL_421863	6.347699999999997E-4	8.617999999999972E-5	63
4640	1.0	EPI_ISL_424136	7.485099999999996E-4	8.617999999999972E-5	63
4640	1.0	EPI_ISL_423892	6.434899999999995E-4	8.617999999999972E-5	63
4640	1.0	EPI_ISL_420479	6.413099999999995E-4	8.617999999999972E-5	63
4640	1.0	EPI_ISL_423147	7.318499999999992E-4	8.617999999999972E-5	63
4640	1.0	EPI_ISL_421772	6.522099999999993E-4	8.617999999999972E-5	63
4640	1.0	EPI_ISL_420709	6.913499999999993E-4	8.617999999999972E-5	63
4640	1.0	EPI_ISL_421788	6.700599999999997E-4	8.617999999999972E-5	63
4640	1.0	EPI_ISL_416517	6.543899999999993E-4	8.617999999999972E-5	63
4640	1.0	EPI_ISL_421894	6.909399999999993E-4	8.617999999999972E-5	63
4640	1.0	EPI_ISL_421862	6.522099999999993E-4	8.617999999999972E-5	63
4640	1.0	EPI_ISL_423692	6.604199999999999E-4	8.617999999999972E-5	63
4640	1.0	EPI_ISL_421815	6.991499999999998E-4	8.617999999999972E-5	63
4640	1.0	EPI_ISL_423484	6.669599999999998E-4	8.617999999999972E-5	63
4640	1.0	EPI_ISL_423634	6.778599999999995E-4	8.617999999999972E-5	63
4640	1.0	EPI_ISL_421799	6.478499999999994E-4	8.617999999999972E-5	63
4640	1.0	EPI_ISL_421912	6.434899999999995E-4	8.617999999999972E-5	63
4640	1.0	EPI_ISL_420635	6.734999999999996E-4	8.617999999999972E-5	63
4640	1.0	EPI_ISL_425273	7.263099999999993E-4	8.617999999999972E-5	63
4640	1.0	EPI_ISL_420478	6.500299999999994E-4	8.617999999999972E-5	63
4640	1.0	EPI_ISL_424012	7.01839999999999E-4	8.617999999999972E-5	63
4640	1.0	EPI_ISL_420727	7.034999999999998E-4	8.617999999999972E-5	63
4640	1.0	EPI_ISL_421774	7.013299999999998E-4	8.617999999999972E-5	63
4640	1.0	EPI_ISL_423260	6.691399999999997E-4	8.617999999999972E-5	63

bioRxiv preprint doi: https://doi.org/10.1101/2020.11.06.370999; this version posted November 6, 2020. The copyright holder for this preprint (which was not certified by peer review) is the author/funder, who has granted bioRxiv a license to display the preprint in perpetuity. It is made available under aCC-BY 4.0 International license.

Table S4: Clusters identified using a genetic distance threshold within 1% of the distribution of patristic distances within the entire tree. The minimum percentile threshold that maximized the number of clusters was chosen as the optimal threshold by performing multiple clustering runs on randomly sampled patristic distance distributions (1 million for each run) in Phylopart v2 (*46*).

clustername	bootstrap	leafname	branchPath	medianOfDistances	sequencesperCluster
4640	1.0	EPI_ISL_421895	6.843999999999994E-4	8.617999999999972E-5	63
4640	1.0	EPI_ISL_421775	6.947899999999999E-4	8.617999999999972E-5	63
4640	1.0	EPI_ISL_421779	7.01839999999999E-4	8.617999999999972E-5	63
4640	1.0	EPI_ISL_423296	6.711999999999997E-4	8.617999999999972E-5	63
4640	1.0	EPI_ISL_423987	6.745599999999997E-4	8.617999999999972E-5	63
4640	1.0	EPI_ISL_423446	6.369499999999996E-4	8.617999999999972E-5	63
4640	1.0	EPI_ISL_423265	6.800399999999995E-4	8.617999999999972E-5	63
4640	1.0	EPI_ISL_419998	7.215799999999994E-4	8.617999999999972E-5	63
4640	1.0	EPI_ISL_425336	7.162699999999995E-4	8.617999999999972E-5	63
4640	1.0	EPI_ISL_423693	6.999799999999998E-4	8.617999999999972E-5	63
4640	1.0	EPI_ISL_423264	6.931199999999992E-4	8.617999999999972E-5	63
4640	1.0	EPI_ISL_418756	6.522099999999993E-4	8.617999999999972E-5	63
4640	1.0	EPI_ISL_421798	6.778599999999995E-4	8.617999999999972E-5	63
4640	1.0	EPI_ISL_423453	6.865899999999994E-4	8.617999999999972E-5	63
4640	1.0	EPI_ISL_421796	7.013299999999998E-4	8.617999999999972E-5	63
4640	1.0	EPI_ISL_423256	6.865799999999994E-4	8.617999999999972E-5	63
4640	1.0	EPI_ISL_428930	6.522099999999993E-4	8.617999999999972E-5	63
4640	1.0	EPI_ISL_421819	6.369499999999996E-4	8.617999999999972E-5	63
4640	1.0	EPI_ISL_423741	6.996599999999991E-4	8.617999999999972E-5	63
4640	1.0	EPI_ISL_423691	6.625999999999998E-4	8.617999999999972E-5	63
4768	1.0	EPI_ISL_423394	6.990699999999999E-4	6.539999999999975E-6	3
4768	1.0	EPI_ISL_423399	6.990699999999999E-4	6.539999999999975E-6	3
4768	1.0	EPI_ISL_423276	6.968899999999999E-4	6.539999999999975E-6	3
4783	1.0	EPI_ISL_430066	7.034199999999997E-4	6.539999999999758E-6	4
4783	1.0	EPI_ISL_427081	7.034199999999997E-4	6.539999999999758E-6	4
4783	1.0	EPI_ISL_420876	7.034199999999997E-4	6.539999999999758E-6	4

bioRxiv preprint doi: https://doi.org/10.1101/2020.11.06.370999; this version posted November 6, 2020. The copyright holder for this preprint (which was not certified by peer review) is the author/funder, who has granted bioRxiv a license to display the preprint in perpetuity. It is made available under aCC-BY 4.0 International license.

Table S4: Clusters identified using a genetic distance threshold within 1% of the distribution of patristic distances within the entire tree. The minimum percentile threshold that maximized the number of clusters was chosen as the optimal threshold by performing multiple clustering runs on randomly sampled patristic distance distributions (1 million for each run) in Phylopart v2 (*46*).

clustername	bootstrap	leafname	branchPath	medianOfDistances	sequencesperCluster
4783	1.0	EPI_ISL_427088	7.012399999999998E-4	6.539999999999758E-6	4
4791	1.0	EPI_ISL_420045	6.8818E-4	7.312000000000009E-5	2
4791	1.0	EPI_ISL_428358	6.8818E-4	7.312000000000009E-5	2
4794	1.0	EPI_ISL_420034	5.7861E-4	6.539999999999975E-6	3
4794	1.0	EPI_ISL_420839	5.8079E-4	6.539999999999975E-6	3
4794	1.0	EPI_ISL_417440	5.8079E-4	6.539999999999975E-6	3
4803	1.0	EPI_ISL_419929	6.282299999999998E-4	4.582999999999996E-5	15
4803	1.0	EPI_ISL_419177	6.330399999999998E-4	4.582999999999996E-5	15
4803	1.0	EPI_ISL_428712	6.309699999999998E-4	4.582999999999996E-5	15
4803	1.0	EPI_ISL_423345	5.851399999999999E-4	4.582999999999996E-5	15
4803	1.0	EPI_ISL_428962	6.264399999999998E-4	4.582999999999996E-5	15
4803	1.0	EPI_ISL_420606	6.025799999999996E-4	4.582999999999996E-5	15

bioRxiv preprint doi: https://doi.org/10.1101/2020.11.06.370999; this version posted November 6, 2020. The copyright holder for this preprint (which was not certified by peer review) is the author/funder, who has granted bioRxiv a license to display the preprint in perpetuity. It is made available under aCC-BY 4.0 International license.
